# Diffusion imaging of cerebral white matter in persons who stutter: evidence for network-level anomalies

**DOI:** 10.3389/fnhum.2014.00054

**Published:** 2014-02-11

**Authors:** Shanqing Cai, Jason A. Tourville, Deryk S. Beal, Joseph S. Perkell, Frank H. Guenther, Satrajit S. Ghosh

**Affiliations:** ^1^Department of Speech, Language and Hearing Sciences, Sargent College of Health and Rehabilitation Sciences, Boston UniversityBoston, MA, USA; ^2^Department of Communication Sciences and Disorders and the Institute for Stuttering Treatment and Research, Faculty of Rehabilitation Medicine, University of AlbertaEdmonton, AB, Canada; ^3^Department of Brain and Cognitive Sciences, McGovern Institute for Brain Research, Massachusetts Institute of TechnologyCambridge, MA, USA

**Keywords:** stuttering, speech disorders, diffusion tensor imaging, brain connectivity, white matter, probabilistic tractography, regions of interest (ROIs)

## Abstract

Deficits in brain white matter have been a main focus of recent neuroimaging studies on stuttering. However, no prior study has examined brain connectivity on the global level of the cerebral cortex in persons who stutter (PWS). In the current study, we analyzed the results from probabilistic tractography between regions comprising the cortical speech network. An anatomical parcellation scheme was used to define 28 speech production-related ROIs in each hemisphere. We used network-based statistic (NBS) and graph theory to analyze the connectivity patterns obtained from tractography. At the network-level, the probabilistic corticocortical connectivity from the PWS group were significantly weaker than that from persons with fluent speech (PFS). NBS analysis revealed significant components in the bilateral speech networks with negative correlations with stuttering severity. To facilitate comparison with previous studies, we also performed tract-based spatial statistics (TBSS) and regional fractional anisotropy (FA) averaging. Results from tractography, TBSS and regional FA averaging jointly highlight the importance of several regions in the left peri-Rolandic sensorimotor and premotor areas, most notably the left ventral premotor cortex (vPMC) and middle primary motor cortex, in the neuroanatomical basis of stuttering.

## Introduction

Developmental stuttering (for brevity, “stuttering” hereafter) is a disorder of speech production that affects approximately 5–8% of children and 1% of adults (Månsson, 2000; Reilly et al., 2009). Stuttering is characterized by frequent interruption of fluent speech by part-word repetitions, sound prolongations, and silent blocks that impair communication (Bloodstein and Ratner, 2008).

The etiology of stuttering remains unclear and is a topic of active research. However, it is clear that stuttering is associated with abnormalities throughout the neural network for speech motor control (e.g., Beal et al., 2007, 2013; Chang et al., 2008, 2011; Watkins et al., 2008; Chang and Zhu, 2013; Connally et al., in press). Past diffusion MRI studies have provided evidence for white-matter (WM) anomalies in the brains of persons who stutter (PWS). By using the method of tract-based spatial statistics (TBSS, Smith et al., 2006), a few groups have found lower-than-normal fractional anisotropy (FA) in WM underlying various cortical areas and in certain subcortical regions in PWS (Chang et al., 2008; Watkins et al., 2008; Kell et al., 2009; Cykowski et al., 2010; Connally et al., in press; see also the non-TBSS-based study by Sommer et al., 2002). FA is a measure that quantifies the directional selectivity (i.e., anisotropy) of water diffusion in tissue. Evidence has shown that anomalies in WM microstructure (e.g., axon density and degree of myelination) lead to decreases in FA (Beaulieu, 2009).

**Figure 1:**
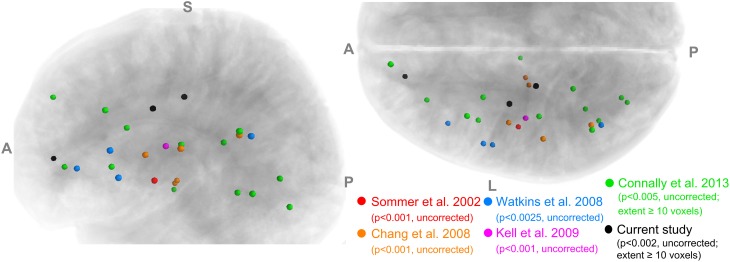
**A summary of voxels with significantly lower FA in PWS than in normal controls reported in five previous studies and the current study**. Only the results in the left hemisphere are shown. **Left panel**: left view; **Right panel**: superior view.

The implicated WM regions varied considerably across the previous studies. For example, in adults who stutter (AWS), Sommer et al. (2002) reported a single locus in the left central operculum, whereas later studies identified greater numbers of loci distributed in the bilateral inferior frontal, peri-Rolandic, inferior frontal and subcortical areas. Cykowski et al. (2010) observed FA reductions in major deep WM tracts. The lack of consistency in spatial localization is illustrated in Figure 1 (see also Figure 2 of Cykowski et al., 2010, p. 1500), which visualizes and compares the left-hemisphere WM clusters found to show lower FA in PWS in five previous studies. Although, as pointed out by Cykowski and colleagues, there is a trend for the loci of FA reductions to cluster in the general regions of the left ventral peri-Rolandic and frontal areas, the detailed location of the FA reductions in those areas was quite variable across studies. In fact, the only region where the loci from different studies came within a distance of 8 mm was the posterior arcuate fasciculus (AF) near the supramarginal gyrus (SMg) (three of five studies: Chang et al., 2008; Watkins et al., 2008; Connally et al., in press), while in other left-hemisphere regions (including the left ventral peri-Rolandic and frontal regions), sites from different studies were at least 11 mm apart.

The diffuse and difficult-to-replicate locations of FA reductions hints at the possibility that WM deficits in stuttering are fundamentally related to, and hence best characterized from, the *connectivity* between specific cortical regions, instead of specific locations in the WM skeleton as extracted by TBSS or other voxel-wise methods. For example, consider the WM fibers that connect two separate cortical regions such as left ventral premotor cortex (vPMC) and SMg, via the AF. Changes in axon fiber microstructure (e.g., demyelination or loss of axons) at different sites along this fiber pathway in different individuals may lead to the same functional deficit, but will appear as FA reductions in varying locations in diffusion-weighted images (DWI) if analyzed with TBSS. Hence, the TBSS analysis would suffer from low contrast-to-noise ratios (difficulty in observing significant effects under corrected statistics, e.g., Connally et al., in press) and between-study discrepancies such as those seen in the previous studies.

Diffusion tractography is a potential method for addressing this limitation. Tractography measures region-to-region WM connectivity. The probabilistic tractography of FMRIB's Diffusion Toolbox (FDT) (Behrens et al., 2003, 2007) has been used to examine structural connectivity between various speech-related regions of the brain in individuals who stutter. Chang et al. (2011) used this method to examine WM connectivity from Brodmann area (BA) 44 in AWS and found the tract densities from left BA 44 to left primary motor cortex (BA 4) and to left premotor cortex (BA 6) were significantly lower in AWS than in fluent controls. Connally et al. (in press) used probabilistic tractography to track AF and corticospinal tract (CST) bilaterally and observed reduced average FA in the left AF in PWS compared to controls. Chang and Zhu (2013) examined WM connectivity in brains of 3–9-year-old-children who stutter (CWS) and found significantly lower-than-normal probabilistic tract density in the basal ganglia-thalamo-cortical loop and in the connections between left inferior frontal gyrus and posterior superior temporal regions. While these prior findings shed important light on the neural underpinnings of this disorder, other important connectivity anomalies may have been missed due to their hypothesis-driven approaches and the limited number of tracts included. In order to obtain a more comprehensive characterization of the WM structural network deficits in stuttering, a data-driven, network approach is required.

In the current study, we take the approach of analyzing the WM connectivity between different parts of the cerebral cortex using tractography. Similar approaches have been applied to other neurological diseases and brain disorders. For example, Zalesky et al. (2010, 2011) proposed and used the network-based statistic (NBS) method to study schizophrenia. They observed a connected subnetwork (i.e., component) that showed reduced connectivity in the patients compared to normal controls. To our knowledge, diffusion tractography on the scale of the speech production and perception network and graph theory-based analyses have not been applied to brain connectivity in stuttering.

We focus on connectivity among a set of speech-related cortical regions in PWS compared to matched controls. These regions are defined through a novel semi-automatic anatomical parcellation scheme called *SpeechLabel* (section SpeechLabel: Anatomically Based Parcellation of the Cerebral Cortex with a Focus on the Speech Network). We also perform correlations between the tractography measures and stuttering severity, so as to identify possible structural bases for severity variation among PWS. In addition, we present results from TBSS, region-average FA, as well as morphometric analyses of the speech-related regions of interest (ROIs), in order to examine the relations of these FA and morphometric results to tractography results and to facilitate comparison with previous studies.

## Methods

### Participants

The procedures of this study were approved by the M.I.T. Committee on the Use of Humans as Experimental Subjects. Twenty right-handed PWS (5 female, age range: 18–47, median: 25.5) and 18 right-handed persons with fluent speech (PFS; 4 female, age range: 19–43, median: 24.5) participated in this study. The median and distribution of the ages were both well matched between the PWS and PFS groups (Wilcoxon rank-sum test: *p* > 0.81; Kolmogorov–Smirnov test: *p* > 0.99). All participants were native speakers of American English.

A speech-language pathologist (SLP, Deryk S. Beal) interviewed all participants to confirm the diagnosis of persistent developmental stuttering in the PWS and to confirm normal speech production in the PFS. The control participants reported no history of stuttering or other fluency disorders. Additional exclusion criteria included: (a) history of speech or language disorders (apart from stuttering for the PWS group), (b) history of neurological or movement disorders, (c) claustrophobia that contraindicates MRI, or (d) current use of medications that may have substantial effects on brain activities and performance on speech tasks.

The stuttering severity of each PWS was assessed using the Stuttering Severity Instrument 4 (SSI-4; Riley, 2008). Each PWS was video-recorded while reading aloud, conversing with the SLP, and speaking on the telephone. The SLP rated the frequency and durations of the stuttering events and the presence of physical concomitants that accompanied the moments of dysfluency, based on which the SSI-4 of the PWS was derived. The stuttering severity in the PWS group ranged from 13 to 43, with a median of 26.0 and an interquartile range of 11.2. Five of the 20 PWS were categorized as “very mild” (SSI-4 score <17), four as “mild” (18–24), six as “moderate” (25–31), three as “severe” (32–36), and two as “very severe” (37–46).

### MR image acquisition

#### Scanner and T1-weighted high-resolution structural scan

MRI images were acquired using a Siemens Magnetom Trio 3-Tesla scanner equipped with a 32-channels head coil at M.I.T. Martinos Center for Biomedical Imaging. A high-resolution T1-weighted image of the head was collected using the magnetization-prepared rapid acquisition gradient echo (MPRAGE) sequence (*TR* = 2530 ms; *TE* = 1.64–7.22 ms; *TI* = 1400 ms; flip angle = 7°; 1 × 1 × 1-mm^3^ isotropic voxels; matrix size: 256 × 256; 172 slices).

#### Diffusion-weighted imaging

A spin-echo echo-planar sequence (*TR* = 8420 ms; *TE* = 84 ms; 2 × 2 × 2-mm^3^ isotropic voxels; matrix size: 128 × 128; 67 slices) was used to acquire whole-brain DWI. Ten no-diffusion images (*b* = 0) were acquired at the beginning of the diffusion sequence. Sixty gradient orientations were applied at *b* = 700 s/mm^2^.

### Speechlabel: anatomically based parcellation of the cerebral cortex with a focus on the speech network

**Figure 2:**
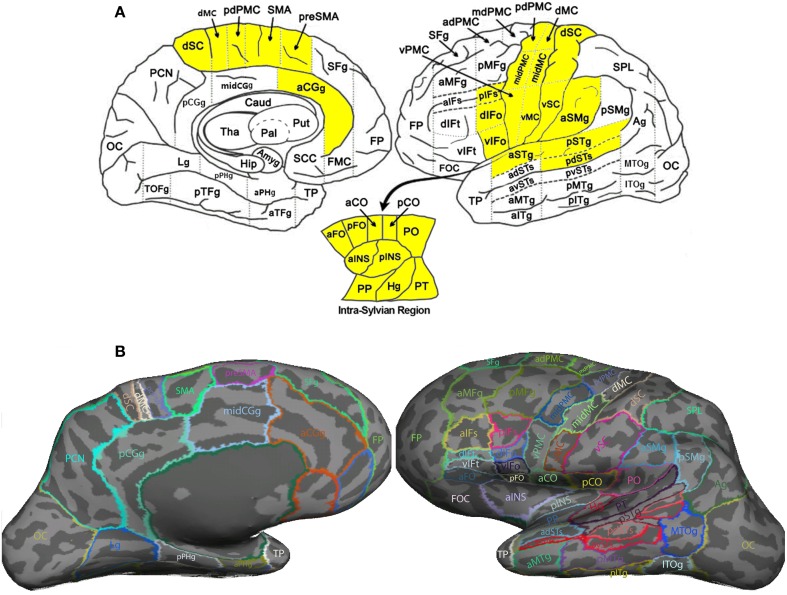
**SpeechLabel parcellation of the cerebral cortex. (A)** A schematic diagram showing the locations of all SpeechLabel ROIs in the left hemisphere. The yellow-highlighted ROIs belong to the speech network subset of SpeechLabel. The same parcellation paradigm and speech subset applies to the right hemisphere, which is not shown in this figure. The left and right parts show the medial and lateral surfaces of the hemisphere, respectively. The intra-Sylvian (opercular) region is shown in the break-out plot at the bottom. **(B)** The result of applying SpeechLabel and subsequent manual correction on the left hemisphere of an example subject, rendered on inflated cortical surface.

Image segmentation, cortical surface reconstruction and surface-based coregistration were performed on the high-resolution T1-weighted image using *FreeSurfer* 5.0.0 (http://surfer.nmr.mgh.harvard.edu/; Fischl, 2012). Each cortical hemisphere was then parcellated into 63 ROIs according to the *SpeechLabel* cortical labeling system. This new parcellation extends the cortical parcellation paradigm of Nieto-Castanon et al. (2003) by including finer-scale subdivisions of the cortical regions involved in speech compared to the default protocols offered by FreeSurfer (see Figure 2).

Figure 2A illustrates the full set of 63 SpeechLabel ROIs in the left hemisphere. The parcellation in the right hemisphere is symmetrical to the left hemisphere. In Figure 2B, the SpeechLabel results (after manual corrections) on an example participant is illustrated on the inflated cortical surface. Among these 63 ROIs, a subset of 28 belonged to the *speech network*, i.e., the set of regions that are functionally activated during speech production (yellow-colored ROIs in Figure 2A). See Supplemental Materials for details on the cortical labeling process and the determination of the speech-network subset of ROIs.

### Preprocessing of diffusion-weighted images

As a first step in the DWI data processing, *DTIPrep* (Liu et al., 2010) was used to perform automated quality control and artifact correction. DWI image frames with excessive head motion- or intensity-related artifacts were excluded from further analysis. The median number of excluded frames per participant in the PFS and PWS groups were 2 and 0.5 (Interquartile ranges: 2, 2), respectively. The data from one control participant was entirely excluded because too many bad gradient directions were found in the DWI data of that subject, leading to group sizes of 20 PWS and 17 PFS in the diffusion MRI data set. Eddy-current correction was then performed on the quality-controlled DWI data.

### Tract-based spatial statistics

For each participant, the FA image computed with FreeSurfer *dtifit* was used as input to TBSS (Smith et al., 2006). We used a FA threshold of 0.2 to exclude non-WM from analysis. Between-group comparisons and correlations with SSI-4 severity scores were performed using general linear models (GLMs) along the WM skeletons.

No clusters of voxels on the WM skeleton survived correction for multiple comparisons under threshold-free cluster enhancement (TFCE; Smith and Nichols, 2009). Therefore, a less stringent correction, consisting of voxel-wise uncorrected, two-tailed *p* < 0.002 and cluster size threshold of 10 voxels (similar to the thresholds in Chang et al., 2008; Watkins et al., 2008; Connally et al., in press), was used for identifying significant between-group differences.

Significant clusters were labeled by the Johns Hopkins University-International Consortium for Brain Mapping (JHU-ICBM) WM (from FSL version 5.0). However, if a JHU-ICBM label for the cluster was unavailable, a probabilistic map of SpeechLabel ROIs, obtained from all of the participants in the current study and transformed into the same diffusion space as the TBSS statistical results, was used to label the cluster.

### Average fractional anisotropy values in regions of interest

TBSS uses the maximum FA along directions perpendicular to points on the skeleton to project individual subjects' FA values (Smith et al., 2006). This ignores non-maximum FA values, which may play as important a role in WM structural connectivity as the maximum-FA voxels. Furthermore, the skeleton projection in TBSS also leads to a partial loss of information about the actual location of the significant WM differences or correlations. An approach that we used to overcome these limitations of TBSS was to compute the FA values in the WM ROIs directly underlying the SpeechLabel gray-matter (GM) ROIs. Such WM ROIs, which can be generated with FreeSurfer's *mri_aparc2aseg* program, are restricted to relatively shallow WM. Hence FA analyses based on these WM ROIs can be regarded as complementary to TBSS.

Average FA values were computed in the SpeechLabel ROIs using 2-mm deep WM ROIs, yielding 63 mean FA values per hemisphere and 126 per participant. These regional average FAs were compared between groups correlated with stuttering severity. The same approaches are used to analyze other tensor measures, including mean diffusivity (MD) and axial diffusivity (AD).

### Probabilistic tractography

The DWI images processed by DTIPrep were used in Bayesian estimation of the diffusion-signal model parameters (FDT *bedpostx*). The sampling results from bedpostx were used for probabilistic tractography (probtrackx2, Behrens et al., 2003, 2007). The following setting for probtrackx2 was used: number of samples = 5000, number of steps per sample = 2000, step length = 0.5, loop check = True, curvature threshold = 0.2, correct path distribution for pathway length = True.

In this study, we examined only the connectivity patterns among the speech-network ROIs of the same hemisphere. For each hemisphere of the cerebral cortex, each of the 28 speech-network ROIs was used as the seed for probtrackx2. Using the tract density image (*fdt_paths*) generated by each run of probtrackx2, the mean tract density inside all 28 ipsilateral speech-network ROIs were computed using *fslstats*. For normalization, the mean tract density values were divided by the number of voxels in the seed mask. By repeating the above procedures overall all 28 speech-related ROIs, an intra-hemispheric speech-network connectivity matrix *M*_0_ was computed for the participant. This matrix is generally asymmetric, due to the asymmetry in the probtrackx2 results. However, diffusion weighted images contain no intrinsic information about the direction of water diffusion. The asymmetry in the tractography results is purely an artifact of the tract-tracing algorithms. Therefore, we defined the symmetric intra-hemispheric connectivity matrix as *M* = $\frac{1}{2}$ (*M*_0_ + *M*^*T*^_0_).

Commissural (inter-hemispheric) connections among the speech-network ROIs were also examined with the same procedure. However, for commissural tractography, the corpus callosum mask was used as a compulsory waypoint in *probtrackx2*. The commissural connectivity matrix was 28 × 28 in size. Unlike the intra-hemispheric connectivity matrices, the commissural matrix was asymmetric. Combining the asymmetric commissural matrix and the two symmetric intra-hemispheric matrices, we could obtain the full 56 × 56 bilateral connectivity matrix, which captured the full ROI-ROI connectivity in the left and right cortical speech networks.

### Statistical and graph-theory analyses of tractography results

For each connection in the unilateral speech network, the mean normalized tract densities in the PWS and PFS groups were compared using the non-parametric Wilcoxon rank-sum test. We also used non-parametric Spearman's correlation to assess the relations between tract densities and SSI-4 (severity) scores in the PWS group. Since each intra-hemispheric connectivity matrix has $\left(\begin{array}{c} 2\\ 28 \end{array}\right)$ = 378 unique connections, the total number of rank-sum tests or Spearman's correlations conducted was 378 × 2 = 756 for each hemisphere.

The same group comparison and severity correlation analyses were performed on the full bilateral connectivity matrix, which consisted of $\left(\begin{array}{c} 2\\ 56 \end{array}\right)$ = 1540 unique connections. Note that 784 (=28^2^) of the 1540 connections were commissural connections, whereas the remaining 756 connections were identical to those in the intra-hemispheric matrices.

Two methods, both based on random permutation, were used to control for family-wise error (FWE). The first method was the NBS proposed by Zalesky et al. (2010). In this method, the results of the statistical tests on individual elements of the connectivity matrix are thresholded to form a set of supra-threshold edges. The sizes of the sets of connected supra-threshold edges (i.e., *components*, in graph-theory terminology) are assessed against an estimated null distribution of maximum component size constructed through random permutation, so that a corrected *p*-value can be assigned to each supra-threshold component.

This second approach was a new method inspired by NBS. It followed the random permutation framework of NBS, but instead of examining size of the network components, it assessed the significance of the bias toward a certain sign of difference (i.e., negative difference: PWS < PFS; or positive difference: PWS > PFS) over all elements of the connectivity matrix. We performed random permutation to calculate significance of the deviation of the *ratio* of positive and negative differences from the null hypothesis of equal likelihood of the two signs, which is a reasonable hypothesis under the assumption of no between-group difference. Laplacian correction was applied to the counts to avoid division by zero. The number of iterations used in this test was 10,000. Note that the binomial test, albeit simpler, is inappropriate here, because tract-density values cannot be assumed to be independent between elements of a connectivity matrix, many of which share the same seeds or targets.

**Figure 3:**
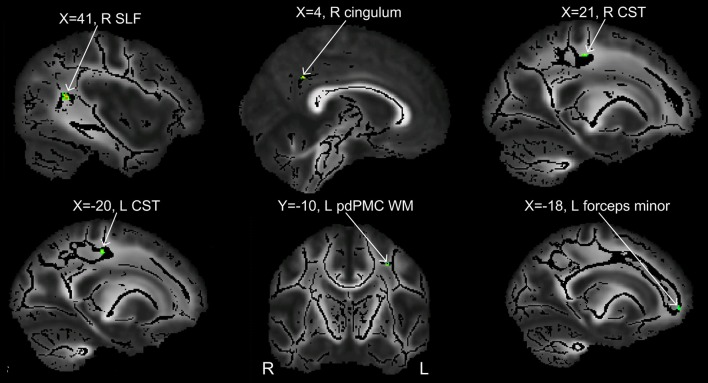
**Clusters showing significant between-group difference in FA values (*p* < 0.002, cluster size >10 voxels)**. All the six clusters in this figure contain differences in the direction of PWS < PFS.

We used an alpha value of 0.05 and one-tailed statistics for obtaining the *p*-values from the random permutation results (including NBS). For the between-group comparison, the tail was selected such that the alternative hypotheses were that there are more negative (PWS < PFS) differences in the connectivity matrix than there are positive ones (for the permutation-based ratio test), that PWS have lower-than-normal tract densities in the connectivity matrix (for the NBS test on between-group difference), and that the correlation between tract density and stuttering severity is negative (for the NBS test on tractography-severity correlation). The use of one-tailed *p*-values in these random permutation test is justified by the observation of predominantly negative (PWS < PFS) differences in FA in the previous diffusion imaging studies on stuttering (Sommer et al., 2002; Chang et al., 2008; Watkins et al., 2008; Cykowski et al., 2010; Connally et al., in press) and the current study (e.g., see Table 1 and Figure 3). The permutation-based statistical methods were applied to the speech networks in the two hemispheres separately.

**Table 1:** **TBSS results: clusters of voxels with significant between-group differences in fractional anisotropy**.

**Cluster location**	**MNI coordinates**	**Cluster extent (mm^3^)**	**Peak *t*-value**	**Peak cohen's *d***
**PWS < PFS**				
R superior longitudinal fasciculus in the white matter underlying R pvSTs	(41, −46, 13)	26	5.37	1.85
R cingulum near R pCGg	(4, −48, 34)	10	4.92	1.70
R corticospinal tract	(21, −23, 47)	16	4.55	1.57
L corticospinal tract	(−20, −23, 46)	20	4.51	1.56
WM underlying L pdPMC	(−29, −10, 43)	11	4.38	1.51
L forceps minor	(−18, 51, 0)	12	3.88	1.34
**PWS > PFS**				
(None)				

### Graph theory analysis of connectivity matrices

We used graph theory to analyze the network properties of WM connection in the speech network. The symmetric connectivity matrix computed for each cerebral hemisphere is a weighted undirected graph. In graph theory, each node in a weighted undirected graph can be characterized by three basic measures: (a) *strength*, (b) *betweenness centrality*, and (c) *clustering coefficient*. The strength of a node is defined as the sum of the weights between the node and all other nodes; it characterizes the interconnectedness of the node in the graph. Betweenness centrality is a global measure of the importance of a node in the network; it is defined as the fraction of shortest path between all nodes of the graph (less the one in question) that pass through the node. Nodes with high betweenness centrality are considered *hubs* in the network. Clustering coefficient is a measure of the degree to which the neighbors of a node are directly connected to each other. It is related to the complexity of local interactions the network can support. Apart from these node-level graph-theory measures, we also computed the *weighted global efficiency* of the WM networks. Weighted global efficiency is inversely proportional to the average shortest path lengths between all pairs of nodes in the network. It quantifies the efficiency with which information can flow among the nodes of the network. We used Brain Connectivity Toolbox (Rubinov and Sporns, 2010) for graph theory analyses.

## Results

We completed TBSS, regional FA and tractography connectivity analyses to determine the local and network properties of WM structural connectivity in PWS. Below we describe the results from the TBSS analysis first, for the purpose of comparison to previous literature. After presenting some findings on the basic morphometric properties of the speech-related ROIs, we extend our analysis to the surface-based WM FA within the speech network. Then, we present results from our tractography and graph theory analyses, providing insight into the connectivity patterns of the neural network for speech production in PWS.

### TBSS results

In order to compare the current data with results from previous studies and to examine the relations between localized FA deficits and network-level deficits, we performed TBSS on the diffusion data from the 20 PWS and 17 matched fluent controls. Six clusters showed significant differences in FA at the threshold of voxel-wise uncorrected *p* < 0.002 and cluster size = 10 voxels, i.e., a threshold comparable to those used in prior TBSS studies on stuttering (see summary in Figure 1). All six clusters showed mean FA values lower in the PWS than in the controls (See Table 1 and Figure 3). Three significant clusters were found in the left hemisphere, distributed in the corticospinal tract, the WM under the posterior dorsal premotor cortex (pdPMC) and in the forceps minor. The right hemisphere also contained three significant clusters, located in the superior longitudinal fasciculus close to the posterior ventral superior temporal sulcus (pvSTs), the cingulum, and the corticospinal tract, respectively.

In addition to the between-group comparison, we performed correlation with SSI-4 scores in the TBSS analysis. The correlation did not reveal any significant voxel clusters under the same liberal threshold as above.

### Morphometric analysis of speech-related cortical regions

Each cerebral hemisphere was parcellated into 63 ROIs, including 28 speech-network ROIs, according to the *SpeechLabel* parcellation paradigm (section SpeechLabel: Anatomically Based Parcellation of the Cerebral Cortex with a Focus on the Speech Network). As areas and thicknesses are basic morphometric properties of cortical regions that may influence or be related to WM connectivity, we briefly present regional morphometry findings before showing results from the ROI-based WM analyses.

We performed between-group comparison of the surface area and cortical thickness for each of the 56 speech-network ROIs in both hemispheres. Under the uncorrected threshold of *p* < 0.05 (two-tailed), the following three significant differences were found: (a) the cortical thickness of left anterior frontal operculum (aFO) was lower in PWS compared to controls; (b) the left aFO showed greater surface area in PWS than in controls; and (c) the left dorsal inferior frontal gyrus pars opercularis (dIFo) showed smaller surface area in PWS compared to controls. When the left dorsal and ventral inferior frontal gyrus pars opercularis (dIFo and vIFo) were considered as a whole, i.e., the left inferior frontal gyrus pars opercularis (IFo) or approximately BA 44, the surface area also showed a significant smaller mean value in PWS than in PFS (*p* = 0.038; mean ± *SD*: PWS: 915.1 ± 248.1 mm^2^; PFS: 1079.6 ± 205.8 mm^2^), which was consistent with the voxel-based morphometry (VBM) results of Kell et al. (2009) in AWS. The between-group difference in the surface area of the left dIFo and cortical thickness of left aFO were largely consistent with the VBM findings by Chang et al. (2008) and Beal et al. (2013) in CWS. More thorough and detailed morphometric analyses of the SpeechLabel ROIs are beyond the scope of this article.

### Regional average fractional anisotropy values

**Figure 4:**
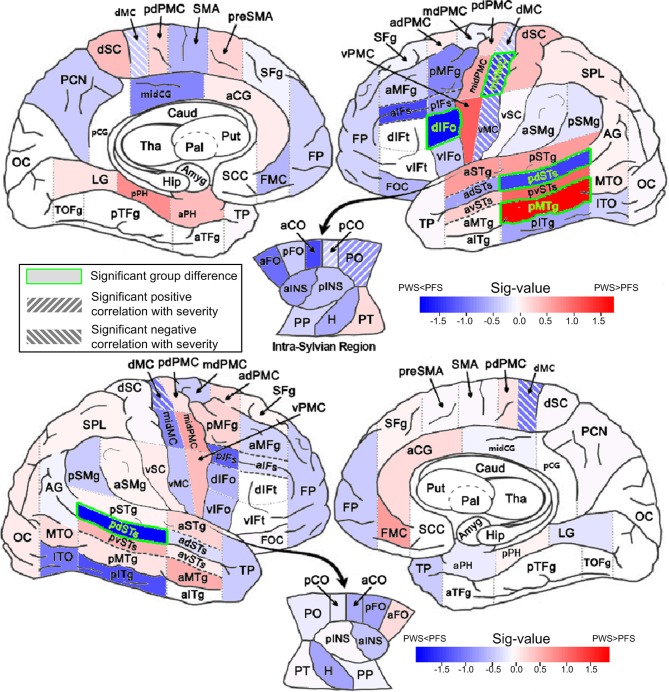
**Between-group comparisons of mean FA in WM ROIs**. The mean FAs were calculated between 0 and 2 mm below the gray-white boundary. Results from left and right hemisphere are shown in the **top** and **bottom** parts, respectively. The filling colors show the direction and sig-value (defined as −log10p multiplied by the sign of the difference in mean; Red: PWS > PFS; Blue: PWS < PFS). The significant difference (*p* < 0.05, two-tailed, uncorrected) are indicated by the green font and green bounding boxes (left dIFo, midMC, pdSTs, and pMTg; right pdSTs). Significant correlations (positive and negative, *p* < 0.05, two-tailed, uncorrected) are indicated as hashed regions (e.g., left midMC).

Figure 4 shows the results of the ROI-by-ROI between-group comparison of mean FA in 2-mm deep WM regions defined by SpeechLabel, which can be viewed as complementary to the TBSS results as this ROI averaging approach is not restricted to maximum-FA voxels and it provide information about the true anatomical location of the WM regions. Since none of the regions shows between-group differences significant under correction for multiple comparisons (False discovery rate, FDR; Benjamini and Hochberg, 1995), we reported uncorrected results with the caveat that these results should be interpreted with caution.

Four WM regions in the left hemisphere, namely dIFo, middle motor cortex (midMC), posterior dorsal superior temporal sulcus (pdSTs) and posterior middle temporal gyrus (pMTg), showed significant between-group difference in regional average FA (two-tailed *t*-test, *p* < 0.05, uncorrected). The first three ROIs showed lower-than-control FA values in PWS, while the left pMTg, a region outside the speech network, showed greater-than-normal FA in PWS than in controls. The AD in left pdSTs also showed significantly reduction in PWS (Table S5). In the right hemisphere, pdSTs was the only ROI that showed significantly mean FA differences between PWS and PFS (PWS < PFS). Notably, all four WM ROIs with significant negative (PWS < PFS) differences belonged to the speech network.

Significant positive correlations between regional average FA and the SSI-4 severity score were found in the left posterior central operculum (pCO) and parietal operculum (PO) (linear correlation, *p* < 0.05, two-tailed, uncorrected). The ROIs that showed significant negative correlations (under the same p threshold) were all in the bilateral precentral primary motor areas, which included left ventral, middle and dorsal motor cortex (vMC, midMC, and dMC), in addition to the dMC on the right (also see Table S3 in Supplemental Materials). It is noteworthy that the left midMC, a part of the speech network, was the only region that showed both a significant difference in FA value (PWS < PFS) and a significant (negative) correlation with the severity score. The AD in this region also showed significant negative correlation with severity (Table S4). The full set of results of analyses on the other basic tensor measures (MD, AD, and RD) can be found in the Supplemental Materials (Tables S4–S6).

### Group differences in speech-network tractography results: intra-hemisphere connections

**Figure 5:**
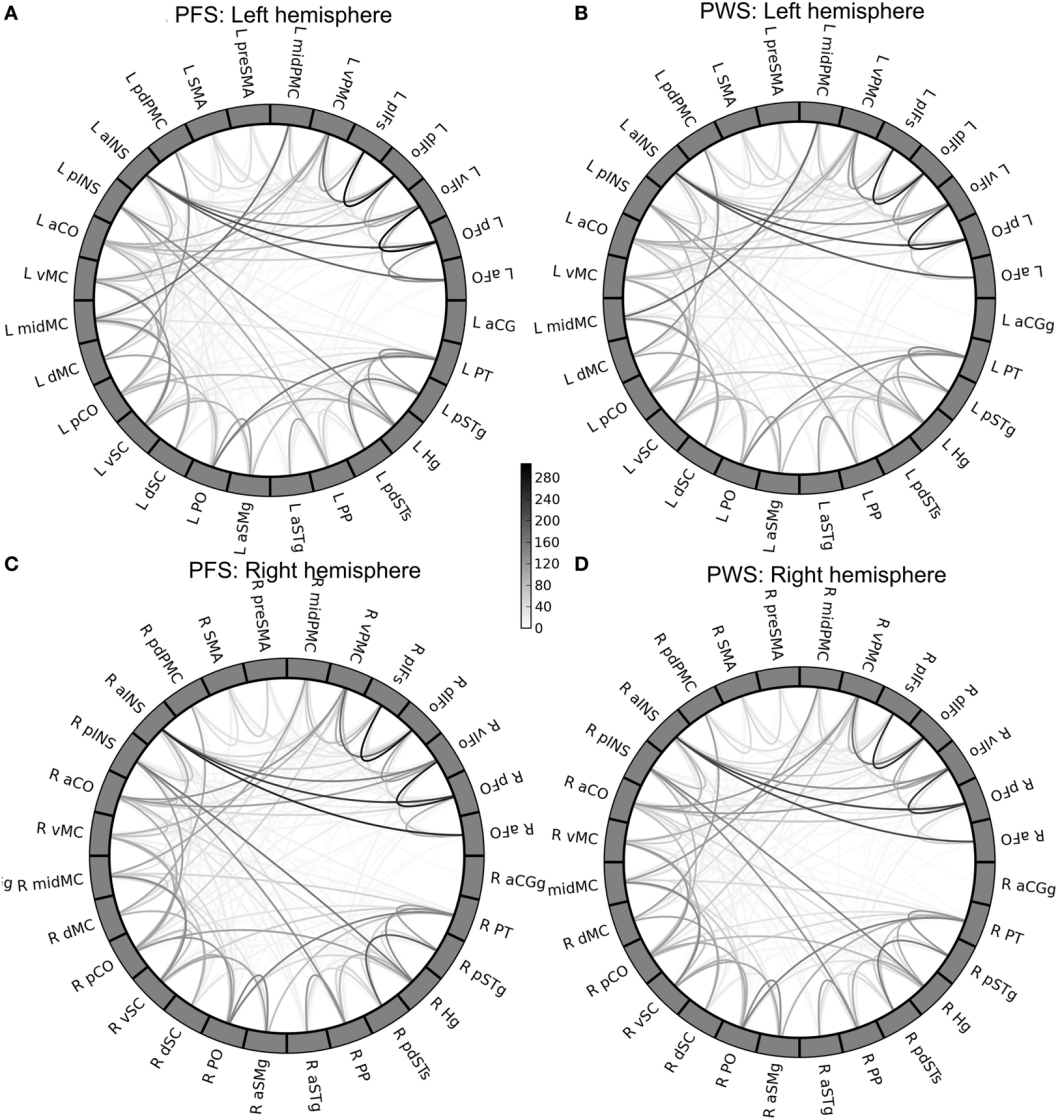
**Group-mean tractography connectivity matrix**. **(A,B)** Show the mean connectivity matrices in the left-hemisphere speech network from the PFS and PWS groups, respectively. Similarly, **(C,D)** show the right-hemisphere speech-network mean connectivity matrices of the PFS and PWS groups, respectively. The ROIs in each panel are ordered by the lobe they belong to and by their anterior-to-posterior positions.

The group-mean WM connectivity matrices determined by probabilistic tractography among the 378 connections in the left and right speech network are shown by the circular diagrams in Figure 5. Figures 5A,B of the figure show the average connectivity patterns in the left-hemisphere speech network for the PFS and PWS groups, respectively. Similarly, Figures 5C,D show the average connectivity patterns in the right-hemisphere speech network for the PFS and PWS groups, respectively. The patterns are similar between all four panels: the local connections between nearby ROIs tended to be the strongest connections, while certain longer-range connections can be seen (e.g., between left midMC and left planum temporale, or PT). Subtle differences in certain connections can be seen upon careful examination. For example, the connection between the left vPMC and left ventral somatosensory cortex (vSC) was on average much weaker (i.e., lighter in grayscale as shown in Figure 5) in the PWS than in the PFS.

**Figure 6:**
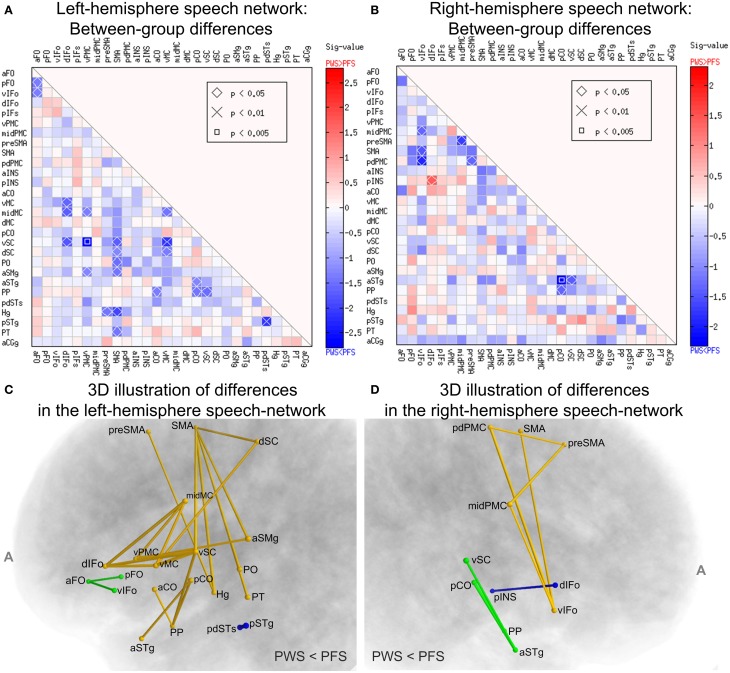
**Between-group comparisons of WM connectivity in the speech network. (A,B)** Illustrate the results from the left and right hemispheres, respectively. Each cell corresponds to the tractography connectivity strength between two SpeechLabel ROIs. In each panel, only the lower triangle is shown due to the symmetry of the connectivity matrix. The sig-values (defined as –log10p multiplied by the sign of the difference) are indicated by the cell's filling colors. Red and blue indicate differences in the directions of PWS > PFS and PWS < PFS, respectively (see color bars). The cells labeled with the symbols, diamond, ×, and square, correspond to the ROI-ROI connections in which the difference between the PWS and PFS groups reach statistical significance under uncorrected *p* < 0.05, 0.01 and 0.005, respectively. **(C**,**D)** illustrate the set of ROI-ROI connections with lower-than-normal normalized tract density in PWS in a 3D format. **(C,D)** correspond to the left and right speech networks, respectively. The connections are represented as tubes, the cross-sectional radii of which are proportional to –log10p from the Wilcoxon rank-sum test. Different colors illustrate different components (connected sets of edges). The components in this figure were not significant under the NBS analysis.

Systematic comparisons of the WM connectivity strength in the left hemisphere revealed 23 connections with significantly lower normalized tract density in the PWS group than in the PFS group (uncorrected *p* < 0.05, two-tailed; See Figure 6A). Partly due to the large number of comparisons conducted, none of the differences survived correction for multiple comparisons at *FDR* = 0.05. Of the connectivity differences, a few involved the vSC: (a) vSC and vPMC (*p* = 0.0016), (b) vSC and vMC (*p* = 0.0077), the implication of which will be discussed later. Other connectivity differences are distributed diffusely across the network. The ROIs that showed the greatest number of significant connectivity reductions in the PWS group were left SMA (6 connections), vSC (5 connections), and vMC (4 connections). The central role played by these three regions in the subset of connections with reduced connectivity in PWS can be seen more clearly in the 3D diagram in Figure 6C.

Remarkably, none of the connections in the left speech network showed significant difference in the direction of PWS > PFS. This bias toward significant differences in the PWS < PFS direction was assessed by a permutation test and the result was significant (corrected *p* = 0.0368, 10,000 iterations), indicating that this bias was not attributed to chance alone under α = 0.05. Our NBS analysis did not confirm the statistical significance of the components (connected subgraphs) in the set of defective connections in the left hemisphere (*p* = 0.115, edge-wise p threshold = 0.01; 10,000 permutations), partly due to the fragmentation into multiple components in the set of connections (see Figure 6C). Therefore, although the statistical significance of the individual differences in WM connectivity strength did not survive corrections for multiple comparisons, the results nonetheless highlighted a difference in *global* connectivity in the left-hemisphere speech network between PWS and normal speakers.

In contrast, between-group comparisons of the WM connectivity strengths in the right-hemisphere speech network yielded fewer (9, Figures 6B,D) differences and these differences were distributed more evenly in the negative and positive directions (8 vs. 1). In the right hemisphere, the bias toward negative differences did not reach statistical significance under the random permutation test (*p* = 0.22).

### Correlating tractography connectivity with stuttering severity: intra-hemisphere connections

**Figure 7:**
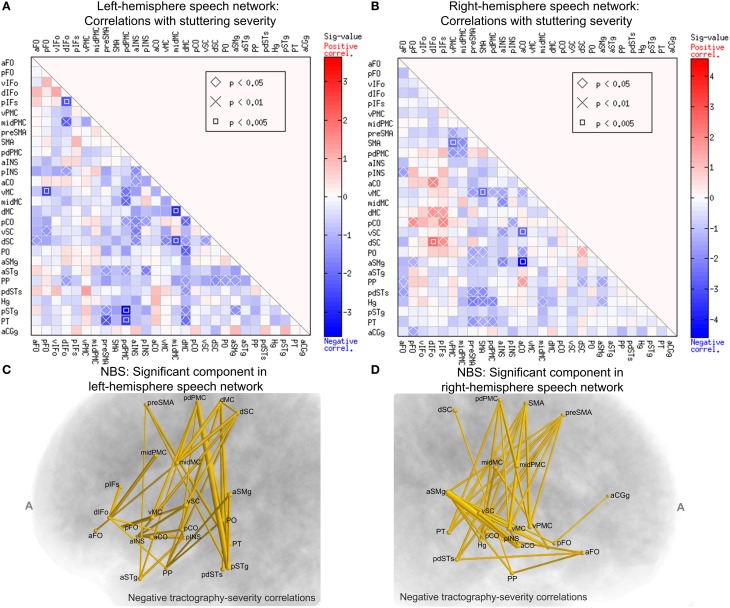
**Spearman's correlations between normalized tract density and SSI-4 severity score in the PWS group**. The format of **(A,B)** are the same as that of Panels **A** and **B** of Figure 6. **(C)** The left-hemisphere component (connected set of edges) with significant negative correlation between normalized tract density and SSI-4 scores (NBS: corrected *p* = 0.0291, one-tailed, 10,000 permutations, edge-wise *p*-value threshold = 0.05; see Methods for details). The edges are represented by tubes with radii that are proportional to −log10p from the correlations. **(D)** The component in the right hemisphere with significant negative correlation with severity (NBS: corrected *p* = 0.0148).

To examine the relation between structural connectivity in the speech network and the severity of stuttering, we performed Spearman's correlations between elements of the WM connectivity matrix of the left-hemisphere speech network and the severity (SSI-4) scores across subjects in the PWS group. The data from all 20 PWS subjects were used this analysis. The marked cells in Figure 7A show the ROI-to-ROI connections with statistically significant correlation between the normalized tract density and the SSI-4 score. The correlation pattern was highly and significantly biased toward the negative direction: all 38 significant correlations were negative (corrected *p* = 0.0126, permutation test, 10,000 iterations).

In the right-hemisphere speech network, the same correlational analysis also revealed a negative-biased distribution of correlations (37 negative vs. 9 positive, Figure 7B), although this bias was not as strong as that seen in the left hemisphere and did not reach significance (*p* = 0.223, permutation test).

We used NBS to assess the significance of the connections correlated with severity and to obtain further protection against family-wise type-I errors under the multiple correlations. Figure 7C shows schematically the component that was significant under the NBS (corrected *p* = 0.0291, one-tailed, 10,000 permutations). This component consisted of 21 nodes and 38 edges. Anatomically, this component is distributed widely: it spans the peri-Rolandic (e.g., midMC), lateral premotor, medial premotor, and posterior superior temporal regions. Another NBS analysis, done separately on the right-hemisphere speech network, also revealed a significant component with negative correlations with SSI-4 score. This component involved 37 edges that span several cortical lobes (Figure 7D). The significance of these NBS results was robust under variations of the edge-wise *p*-value threshold.

A closer examination of the significant group differences and the correlations with severity led to the surprising observation that these two sets of results resided in largely non-overlapping sets of connections. Of the 38 left-side connections that showed significant negative correlation with SSI-4, only one (the midMC-vMC connection) showed significantly weaker-than-normal connectivity in PWS. In the right-hemisphere speech network, there is no overlap in the set of connections with significant between-group differences and the set with significant correlation with severity. The implication of this non-overlapping pattern will be discussed later.

### Commissural and bilateral connectivity matrix

**Figure 8:**
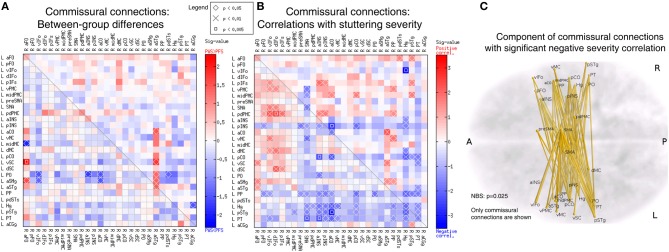
**Analyses of the commissural connectivity matrix. (A)** Between-group differences (same format at Figure 6A). **(B)** Correlation with SSI-4 stuttering severity score (same format as Figure 7A). The 68 negative correlations in this panel form a component that belongs to a larger component in the 56 × 56 bilateral connectivity matrix, which was significant in the NBS analysis (*p* = 0.025). **(C)** 3D illustration of the commissural connection component with significant negative correlation to stuttering severity.

Using the corpus callosum as a compulsory waypoint, we obtained probabilistic tracts between speech-network ROIs of the opposite hemispheres, which formed an asymmetric 28 × 28 commissural connectivity matrix. A few connections in this differed between the PWS and PFS groups at uncorrected *p* < 0.05 (Figure 8A). These differences were relatively fewer compared to the intra-hemispheric results in Figure 6A and not significantly biased in either direction (8 PWS < PFS vs. 7 PWS > PFS; *p* > 0.4). However, a large portion of the matrix showed correlations with the SSI-4 severity score at uncorrected *p* < 0.05 (Figure 8B). Of the significant 97 correlations at uncorrected *p* < 0.05, 35 were positive and 62 were negative (non-significant bias: *p* > 0.2).

All the 62 edges with negative severity correlations formed a component that was nearly significant in the NBS analysis (*p* = 0.052). When the commissural matrix was combined with the two intra-hemispheric matrix to form a 56 × 56 bilateral connectivity matrix, these 68 edges became a part of a large component comprised of 137 edges and 51 nodes located in both hemispheres, which reached significance in the NBS analysis (*p* = 0.025). Examination of this commissural component revealed heavy involvement of the regions in bilateral superior temporal lobe, including bilateral planum polare (PP), Heschl's gyrus (Hg), posterior superior temporal gyrus (pSTg), and PT (Figures 8B,C).

### Graph-theory measures of the connectivity matrices

To analyze the graph properties of the speech networks in PWS and PFS, we used Brain Connectivity Tool box to extract graph-theory measures of the network nodes. As in the regional average FA analyses, the results in this subsection are based on uncorrected statistics and hence should be viewed as largely exploratory.

**Figure 9:**
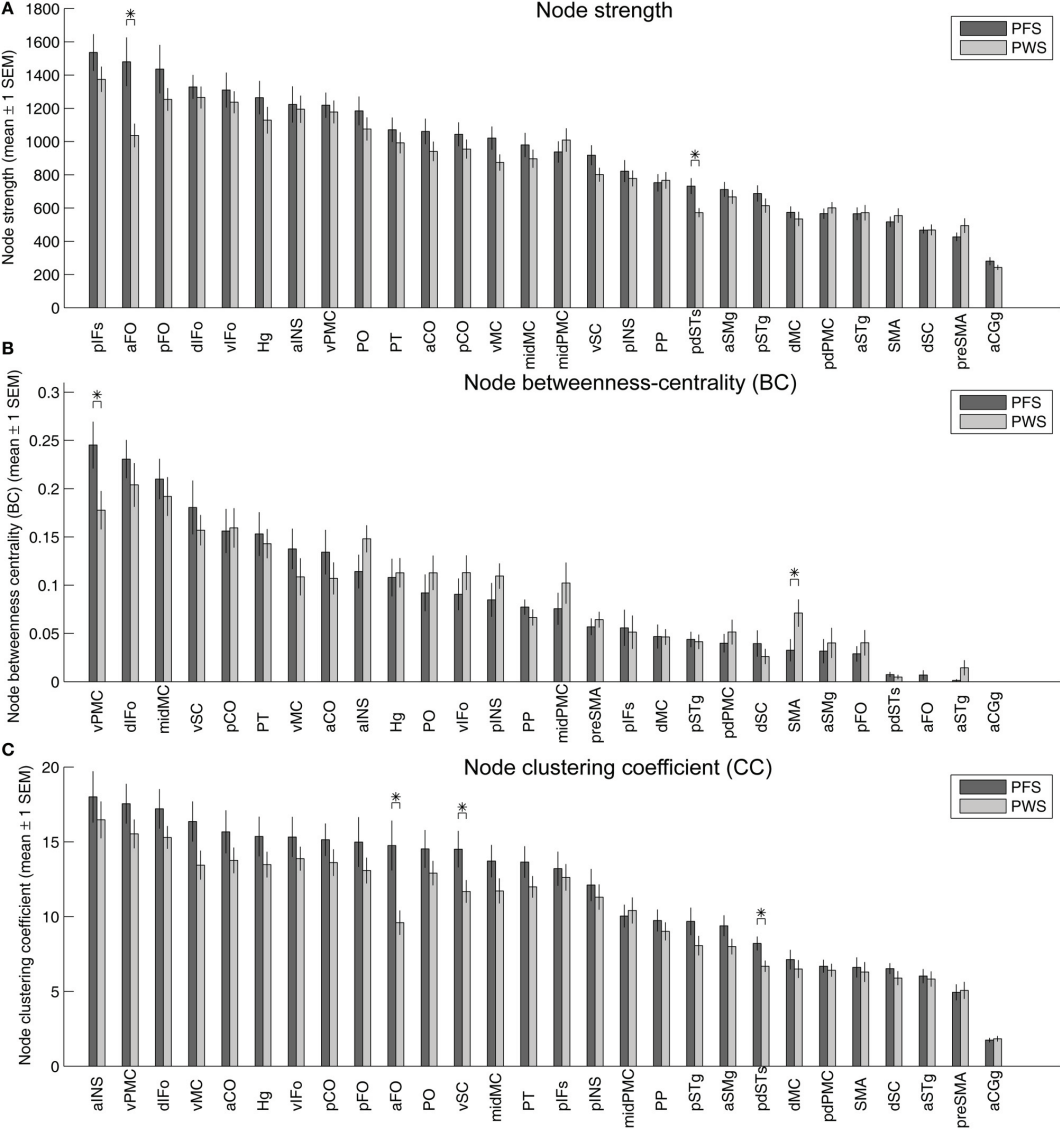
**Strength (A), betweenness centrality (B), and clustering coefficients (C) of the 28 nodes in the left-hemisphere speech network**. In each panel, the gray and red bars show the mean ± 1 s.e.m. of the values in the PFS and PWS groups, respectively. The nodes are sorted by descending order of mean values in the PFS group. Asterisks indicate significant difference between groups at *p* < 0.05 (two-tailed *t*-test, uncorrected).

Figure 9A illustrates the strengths (summed normalized tract density) of the nodes in the left-hemisphere speech network, with the nodes sorted in descending order of the mean strengths in the PFS group. Two of the nodes showed significantly different mean strength between PWS and PFS. The mean strength of both nodes, the left aFO and pdSTs, were lower in PWS than in controls. The lower-than-normal strength in left pdSTs was consistent with the finding of decreased average FA in the 2-mm-deep WM underlying this region (Figure 4). These group differences in the strength of the left aFO and pdSTs held when the bilateral connectivity matrix was examined (not shown).

Under the same statistical threshold, no nodes in the right-hemisphere speech network showed significant differences in strength between PWS and controls.

Betweenness centrality, defined as the fraction of shortest paths between all pairs of nodes that pass through a given node, quantifies the importance of the node in facilitating efficient node-to-node communication in the network. In the left-hemisphere speech network of the PFS group, the node with the highest average betweenness centrality was the vPMC, indicating that this region serves as an important structural hub in the left-hemisphere speech network. This hub region showed significantly lower centrality in the PWS compared to controls (*p* = 0.037; Figure 9B). Another area that showed significant difference between PWS and PFS was the left SMA (PWS > PFS; *p* = 0.048, two-tailed; Figure 9B).

Three ROIs in the left hemisphere showed significantly lower clustering coefficients in PWS than in PFS: aFO, vSC, and pdSTs (Figure 9C). The left aFO also showed significantly reduced clustering coefficient in the bilateral connectivity matrix (not shown).

In the right-hemisphere speech network, no nodes were found to be significantly different in any of the three node-level graph-theory measures between PWS and PFS under the same statistical threshold as above.

On the network-level, the weighted global efficiency of the two groups did not differ significantly in the left (mean ± 1 s.e.m.: PFS: 0.245 ± 0.011; PWS: 0.235 ± 0.011, *p* > 0.5) or right hemisphere (PFS: 0.258 ± 0.012; PWS: 0.253 ± 0.008; *p* > 0.6). The global efficiency values were not correlated significantly with stuttering severity in the PWS group (left: ρ = −0.37, *p* > 0.1; right: ρ = −0.32, *p* > 0.15). When the left and right hemisphere were examined jointly in the bilateral connectivity matrix, the weighted global efficiency did not differ significantly between groups, either (PFS: 0.168 ± 0.009; PWS: 0.156 ± 0.006; *p* > 0.25). However, the correlation between the bilateral weighted global efficiency and SSI-4 severity scores in the PWS group approached significance (ρ = −0.41, *p* = 0.074).

## Discussion

In this paper, we performed the first examination of global structural connectivity of the cerebral cortex in AWS. To facilitate comparison with prior studies, we also took a comprehensive approach to quantifying the cerebral WM anomalies in PWS. A brief summary of the main findings is presented in Table 2.

**Table 2:** **A brief summary of the main significant findings of this article**.

**Analysis**	**Main findings**
1. TBSS (Table 1 and Figure 3)	Six clusters in both hemispheres with significantly lower FA in PWS than in PFS, with limited consistency with prior studies (uncorrected *p* < 0.002, extend = 10 voxels)
2. Basic morphometry of speech-network ROIs	Cortical surface area PWS < PFS: L dIFo and L IFo (vIFo + dIFo) Cortical thickness PWS < PFS: L aFO
	Cortical surface area PWS>PFS: L aFO (*p* < 0.05, uncorrected)
3. Regional average FA (Figure 4)	Significantly lower regional average FA in four speech-network ROIs: L dIFo, midMC, pdSTs, and R pdSTs
	Significant negative correlation between regional average FA and SSI-4 severity score in L vMC, midMC, dMC, and right dMC. Significant positive correlation in L pCO and PO (*p* < 0.05, uncorrected)
4. Tractography: Between-group comparison (Figure 6)	In the left hemisphere, 23 connections differed significantly between PWS and PFS. **All 23 differences had the sign of PWS < PFS, which was significantly biased (permutation test**: *p* = **0.0365**)
	The most prominent differences involved connections: vSC-vPMC, vSC-vMC, and pdSTs-pSTg. ROIs with the greatest number of reduced connections: L SMA, vSC, and vMC
5. Tractography: Correlation with stuttering severity (Figure 7)	Left hemisphere: **A significant component of 38 connections negatively correlated with severity (NBS: ***p*** = **0.0291**); significant bias toward negative correlation (permutation test: ***p*** < **0.0126**)**
	Right hemisphere: **A significant component of 37 connections negatively correlated with severity (NBS: *p* = **0.0148**)**
6. Tractography: Graph theory (Figure 8)	Node strength PWS < PFS: L aFO and L pdSTs
	Node betweenness centrality PWS < PFS: L vPMC (top hub in PFS)
	Node betweenness centrality PWS > PFS: L SMA
	Node clustering coefficient PWS < PFS: L aFO, vSC, pdSTs
	(*p* < 0.05, uncorrected)

*The bold and regular faces indicate significance with and without correction for multiple comparisons, respectively*.

The conventional TBSS analysis and regional FA averaging identified loci in both deep and shallow WM with lower-than-normal FA values in PWS and with negative correlations with stuttering severity, most noticeably in the left midMC. For the first time, we obtained a structural connectivity matrix from PWS and fluent controls through diffusion tractography. The results indicated that PWS show subnormal region-region connections in the speech network. In addition, we found a significant bias toward negative (PWS < PFS) differences in the left speech network as a whole. As another important finding, the NBS analysis revealed extensive components in both left and right speech networks with negative correlation with the severity of stuttering. Graph theory analysis highlighted the important role of vPMC in the defective WM connectivity in stuttering, as indicated by the significantly reduced betweenness centrality of this top hub of the speech network. These tractography results, viewed in light of the highly variable local (TBSS and FA) results across studies, support the proposition that WM deficits in stuttering are best characterized by region-region connections involved in speech production.

### Tract-based spatial statistics and regional average fractional anisotropy results: relations to previous literature

Our TBSS results (Figure 3) were largely consistent with previous studies in showing effects predominantly in the direction of PWS < PFS. However, the spatial consistency of these findings with previous ones (Figure 1) was low. For example, like Cykowski et al. (2010), our TBSS analysis failed to show voxels with reduced FA values in PWS in the WM near the left ventral peri-Rolandic region. The clusters in the left CST [(−20, −23, 46)] and left pdPMC [(−29, −10, 43)] that we reported tended to be more superior and medial compared to the previously reported region (see Figure 1).

In contrast to the low consistency between our TBSS results and those of prior studies, by computing the regional average FA values in the 2-mm-deep WM ROIs, we observed significant WM reductions in PWS (under uncorrected *p*-value thresholds) in several regions that were more consistent with previous studies. The lower-than-normal average FA in PWS seen in the left midMC [center-of-gravity MNI coordinates: (−45, −11, 43)] fell into the general area of the left ventral peri-Rolandic region. The second FA reduction was seen in the left dIFo [ROI center-of-gravity MNI coordinates: (−51, 17, 18)], consistent with a voxel cluster in the left inferior frontal gyrus reported by Watkins et al. (2008) to show significantly lower FA in PWS than in fluent controls.

For other significant voxel clusters and regions in which we observed lower-than-normal FA values in the current study, it is difficult to identify matching loci from previous reports. This again highlights the difficulty in reproducing FA deficit findings in PWS (see Introduction), which reflects the possibility that WM deficits in stuttering are best characterized by reduced region-region connectivity, rather than by specific locations along the WM pathways.

### Relations between fractional-anisotropy and tractography findings

To investigate WM tracts at the network-level, we employed the SpeechLabel cortical parcellation scheme to define seeds for tractography and nodes for the graph theory analysis. Comparing the regional-average FA results (Figure 4) and the connectivity findings (Figures 5, 6), one can see some moderate consistencies. For example, for all three left-hemisphere WM ROIs that showed significantly lower-than-normal regional average FA in PWS, including dIFo, midMC, and pdSTs (Figure 4), reduced connectivity weights were seen in the corresponding elements of the connectivity matrix (Figures 6A,C). Hence the FA reductions in the shallow WM underlying these cortical regions may contribute to the decreased tract densities to and from these regions. However, not all connectivity deficits in the tractography results could be accounted for by the regional WM FA reductions. For example, two of the regions with the largest number of weakened connections, namely left SMA and left vMC did not show statistically significant differences in regional average FA between PWS and PFS. This is again consistent with the notion that WM deficits in PWS cannot always be pinpointed to specific spatial locations in the cerebral WM, but are nonetheless specific to the region-region WM connections. For example, it is conceivable that the WM disruption that leads to the defective connection between left vPMC and vSC (Figures 6A,C) may vary in the anterior-posterior position between individual PWS. In some PWS, the loci of disruption may be more anterior, whereas in other PWS, the loci may be more posterior; in still others, the deficit may be distributed along the anterior-posterior axis. Such variation may render the spatial localization of the FA reductions difficult.

### The critical role of left middle motor cortex

The FA in the WM underlying the left midMC showed an especially interesting pattern of both reduction and negative correlation with severity in the PWS, hinting at a central role played by the WM underlying this cortical region in the mechanisms of stuttering. Such a role is corroborated by the presence of both reductions and correlations with severity in the tractography results involving the left midMC (Figures 6C, 7C).

**Figure 10:**
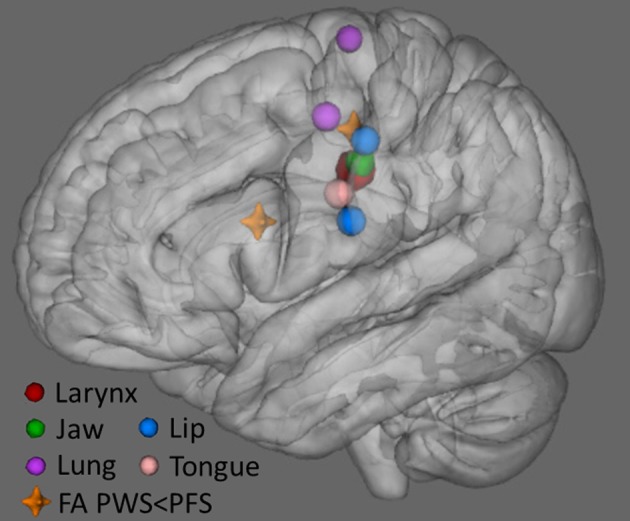
**Locations of lower FA in PWS compared to PFS (orange crosshairs) along with locations of speech articulator representations in sensorimotor cortex (spheres)**. Larynx symbol is enlarged for visibility. The position of the larynx representation is based on Grabski et al. (2012) meta-analysis. Representations of the lung (respiration), lip and tongue are based on Takai et al. (2010) meta-analysis. Jaw representation is based on average of cluster peaks in Onozuka et al. (2002, 2003), and Takahashi et al. (2007), Grabski et al. (2012).

Figure 10 plots the locations in MNI space where we found lower FA in PWS compared to PFS, along with sites representing of the primary articulators for speech as derived from recent meta-analyses of fMRI findings (Takai et al., 2010; Grabski et al., 2012). The location of our L midMC ROI (the more dorsal and posterior of the two locations with lower FA for PWS shown in Figure 9) lies very close to the motor representations of the key articulators for speech, including the lips, jaw, tongue, larynx, and respiratory musculature. This proximity provides further support for a strong link between stuttering and WM integrity beneath midMC.

### White-matter connectivity deficits involving left ventral somatosensory cortex

Another interesting region that emerged from our tractography analysis was the left vSC. This region was observed to be involved in two prominent group differences in normalized tract density. It is also one of the nodes in the left speech network with the largest number of defective connections (Figures 6A,C). In the graph-theory analysis, left vSC showed a significantly reduced clustering coefficient (Figure 9C). Based on the statistical prominence of these connectivity findings, we suggest that future studies should examine more thoroughly the presence and nature of somatosensory deficits in stuttering (e.g., Loucks and De Nil, 2006; Daliri et al., 2013).

The above observations regarding left midMC and vSC were generally consistent with previous findings of FA reductions in the left ventral peri-Rolandic region. They warrant further examination of the WM properties underlying these cortical regions.

### Left inferior frontal gyrus and ventral premotor cortex

Chang et al. (2011) reported significantly lower-than-normal tract density from left BA 44 (inferior frontal gyrus, pars opercularis) to left precentral gyrus using probabilistic tractography in AWS. Their functionally defined BA-44 ROI corresponded roughly to our vIFo and dIFo regions, whereas their left precentral gyrus was divided into the left vMC, midMC, and dMC in the current study. Our finding of lower-than-normal average FA in the dIFo lends some support for WM deficits involving BA 44. Moreover, our tractography results showed that the following connections were significantly weaker in PWS compared to controls: L dIFo-vMC and dIFo-midMC (Figure 6A). In addition, five pathways, namely L dIFo-aCO, dIF o-dMC, vIFO-aCO, vIFo-vMC, and vIFo-midMC, showed lower connectivity in PWS compared to PFS, but did not reach significance at *p* < 0.05, which may be attributable to a lack of power. These tractography differences regarding BA 44-Rolandic connections were all largely consistent with the findings of Chang et al. (2011). Interestingly, among the ROIs that showed anomalies in diffusion-tensor and tractography measures in PWS, the left dIFo was the only one that showed a morphological anomaly (smaller surface area in PWS) and this anomaly was consistent with a prior report (Kell et al., 2009). The coexistence of morphological and connectivity anomalies in this region and its consistency with previous finings hints at a possibly unique and important role played by this regions in the neural mechanisms of stuttering. However, it may be noted that we failed to replicate the BA 44-pSTg connection deficits reported in CWS by Chang and Zhu (2013). This difference in the patterns of WM deficits in CWS and AWS requires more careful examination in the future.

The left vPMC, a region immediately posterior to BA 44, showed significantly weaker-than-normal connectivity with several peri-Rolandic regions in PWS (Figures 6A,C). In addition, analysis of the WM connectivity graphs in the PFS group established the left vPMC as the hub with the highest centrality in the left speech network (Figure 8). This finding was consistent with the critical role played by the left vPMC in the DIVA model of speech motor control (Guenther et al., 2006; Golfinopoulos et al., 2010) and the GODIVA model of speech sequencing (Bohland et al., 2010). In both models, the left vPMC forms part of the hypothesized “speech sound map,” which forms the bridge between the phonological planning in prefrontal cortex and the sensorimotor processes in peri-Rolandic and superior temporal regions. Compromises in this important hub of the speech network, as seen in our between-group comparison of the centrality of left vPMC, should lead to disruptions in the planning and execution of speech (cf. the EXPLAN theory, Howell and Au-Yeung, 2002) and constitute an additional facet of the neural mechanism of stuttering.

In the DIVA model (Guenther et al., 2006; Golfinopoulos et al., 2010), the speech sound map spans the left vPMC and IFo (including vIFo and dIFo). Defective connectivity between these regions and the primary motor cortex (e.g., vMC, midMC; Figures 6A,C) may lead to insufficient readout of the speech motor programs, forming a weakened feedforward pathway for speech motor control, which has been modeled computationally by Civier et al. (2010).

### Relation between connectivity reductions and correlations with severity

In our analysis of the WM connectivity matrices, we performed both between-group comparisons and correlations with stuttering severity. To our knowledge, the finding of the large components of the speech network with negative connectivity-severity correlation (Figure 7) was the first demonstration of brain-behavior correlation in stuttering on such extended scale. However, we found two almost non-overlapping sets of significant connections in the comparison and correlation analyses: the intersection of the two sets contained only one path, between left midMC and left vMC. This result is surprising at first glance, because if the WM connection deficits are assumed to be a causal link in stuttering, one would expect to find greater connectivity deficits in more severe PWS. The absence of such a pattern may be related to spontaneous compensatory mechanisms. The participants used in the current study were adults with persistent developmental stuttering, who had decades of experience coping with this disorder. WM changes have been observed after motor learning (e.g., Scholz et al., 2009; Zatorre et al., 2012). It is possible that the brain of a PWS recruits cortical regions and connections other than the primarily affected ones during speech production to enhance speech fluency, and such long-term use of the alternative regions and connections leads to plastic WM changes, giving rise to the negative correlations between connectivity and severity seen in the current study. If this hypothesis is true, then the brains of CWS, which have less time to implement the compensatory strategies than AWS, should show more overlapping sets of reduced connections and connections negatively correlated with severity. Therefore, performing diffusion tractography analysis in CWS will be an important way to elucidate the relations between WM connections and the onset and development of stuttering (see Chang and Zhu, 2013).

### Left-biased deficits and bilateral correlation with severity

The between-group comparisons of DWI-derived WM measures showed a consistent pattern of left-biased deficits in PWS. For example, three of the four ROIs with lower-than-normal regional average FA values in PWS were in the left hemisphere. Also, the tractography connectivity matrix of the left hemisphere showed 23 between-group differences, exclusively in the negative sign (PWS < PFS), so that a permutation test indicated that this directional bias was significant. No such significant directional bias of group difference was observed in the right hemisphere. In addition, the graph theory analysis showed significant differences in node strength and centrality only in the left hemisphere, but not in the right hemisphere. This overall pattern was largely consistent with reports from previous WM studies, which implicated more WM clusters with reduced FA (Sommer et al., 2002; Chang et al., 2008; Watkins et al., 2008; Kell et al., 2009; Cykowski et al., 2010) and tractography results with reduced tract densities (Chang et al., 2011; Connally et al., in press) in the left hemisphere of PWS than in the right.

Unlike the left-biased between-group differences, negative correlations with stuttering severity were identified in PWS with the NBS analysis in *both* hemispheres (Figure 7) and in the commissural connections between the two hemispheres (Figures 8B,C). Global efficiency, a network-level measure of the interconnectedness of the bilateral speech network, showed a negative correlation with stuttering severity that approached significance (*p* = 0.074). If it can be assumed that the negative tractography-severity correlations are consequences of long-term spontaneous compensation, as discussed in the previous subsection, this observation indicates that bilateral speech-network regions are recruited for the compensation, despite that the primary deficits underlying stuttering may be left hemisphere dominated. This reasoning appears to be consistent with previously observed negative correlations between stuttering severity and functional activations during speech in both left and right cortical regions (e.g., Preibisch et al., 2003; Kell et al., 2009; Toyomura et al., 2011), which may reflect the spontaneous compensatory mechanisms. The heavy involvement of the superior temporal regions in the commissural connections with negative severity correlations (Figures 8B,C) hints at the potential importance of auditory feedback in the mechanisms of persistent developmental stuttering. Functional connectivity between the bilateral temporal lobes is strengthened during articulation under perturbed auditory feedback (Tourville et al., 2008). This WM-severity relation merits further research, especially in the light of the long-known effects of auditory feedback alteration on stuttering (Kalinowski et al., 1993) and the recent findings of subnormal compensation to online auditory feedback manipulation during speech production in AWS (e.g., Cai et al., 2012).

### Limitations

There are some important limitations of this study. First, we used modest subject group sizes, which limited the statistical power of the analyses. Second, although generally good agreement has been found between probabilistic tractography results and axonal pathways reconstructed through tracer injection (Dyrby et al., 2007; Yamada et al., 2008), tractography results may not correspond to true axon fibers in a straightforward manner; differences in tractography connectivity strength may arise from a variety of causes (Jones, 2010). Our findings await replication by future DWI studies and confirmation by studies based on other techniques, such as *post-mortem* histology. Third, as discussed earlier, the current methodology does not allow clear separation of etiological mechanisms from compensatory ones, which requires longitudinal studies. Finally, we focused on WM connectivity within the cerebral cortex. Therefore this study did not provide information about the property of cortical-subcortical WM pathways in PWS (see Chang and Zhu, 2013). There is ample evidence for the involvement of both the basal ganglia (e.g., Alm, 2004; Giraud et al., 2008; Toyomura et al., 2011) and the cerebellum (e.g., De Nil et al., 2001; Lu et al., 2010; Connally et al., in press) in the neural mechanism of stuttering. Results from tractography between the cerebral cortex and the subcortical structures including the basal ganglia and cerebellum will be reported in a future publication.

## Conclusions

Using three different measures of white matter integrity, we identified a number of anomalies in the speech network of PWS. One of the brain regions most implicated as the potential source of stuttering was the left midMC, which showed reductions and negative correlations in both FA and connectivity with other brain regions in the speech network. MidMC contains representations of the key speech articulators, lending further credence to the possibility that impairment to midMC and/or pathways that involve it may be a critical causal link in this disorder. Other anomalies are salient on the level of the network, i.e., above the level of individual regions or connections. Intra-hemisphere connectivity of PWS exhibited global reduction in the left hemisphere. The connectivity in both hemispheres showed extensive negative correlations with stuttering severity. Detailed examination of the tractography results revealed distributed connectivity anomalies involving the left posterior inferior frontal, ventral premotor and peri-Rolandic sensorimotor areas. Jointly or independently, these anomalous WM connections may lead to unreliable readout and execution of speech motor commands, forming the basis of stuttering.

### Conflict of interest statement

The authors declare that the research was conducted in the absence of any commercial or financial relationships that could be construed as a potential conflict of interest.

## Abbreviations

(Table S2 contains a full list of the abbreviations; also refer to Figure 2 and Table S1 for abbreviations of ROI names in SpeechLabel).
AF: arcuate fasciculus
AWS: adults who stutter
BA: Brodmann area
CST: corticospinal tract
CWS: children who stutter
dIFo: inferior frontal gyrus pars opercularis, dorsal division
dMC: (primary) motor cortex, dorsal division
DWI: diffusion weighted imaging
FA: fractional anisotropy
FDR: false discovery rate, FDT, FMRIB's diffusion toolbox
FWE: family-wise error
GM: gray matter
IFo: inferior frontal gyrus, pars opercularis
MNI: Montreal Neurological Institute
NBS: network-based statistic
PFS: person(s) with fluent speech
PWS: person(s) who stutter
ROI: region of interest
SLP: speech language pathologist
SSI: stuttering severity instrument
TE: echo time
TI: inversion time
TFCE: threshold-free cluster enhancement
TR: repetition time
TBSS: tract-based spatial statistic
WM: white matter.

## References

[B1] Alm P. A. (2004). Stuttering and the basal ganglia circuits: a critical review of possible relations. J. Commun. Disord. 37, 325–369 10.1016/j.jcomdis.2004.03.001 15159193

[B2] Beal D. S. Gracco V. L. Brettschneider J. Kroll R. M. De Nil L. F. (2013). A voxel-based morphometry (VBM) analysis of regional grey and white matter volume abnormalities within the speech production network of children who stutter. Cortex 49, 2151–2161 10.1016/j.cortex.2012.08.013 23140891 PMC3617061

[B3] Beal D. S. Gracco V. L. Lafaille S. J. De Nil L. F. (2007). Voxel-based morphometry of auditory and speech-related cortex in stutterers. Neuroreport 18, 1257–1260 10.1097/WNR.0b013e3282202c4d 17632278

[B4] Beaulieu C. (2009). The biological basis of diffusion anisotropy, in Diffusion MRI: From Quantitative Measurement to In-vivo Neuroanatomy, eds Johansen-Berg H. Behrens T. E. (London, UK: Academic Press), 106–123 10.1016/B978-0-12-374709-9.00006-7

[B5] Behrens T. E. Berg H. J. Jbabdi S. Rushworth M. F. Woolrich M. W. (2007). Probabilistic diffusion tractography with multiple fibre orientations: what can we gain? Neuroimage 34, 144–155 10.1016/j.neuroimage.2006.09.018 17070705 PMC7116582

[B6] Behrens T. E. Woolrich M. W. Jenkinson M. Johansen-Berg H. Nunes R. G. Clare S. (2003). Characterization and propagation of uncertainty in diffusion-weighted MR imaging. Magn. Reson. Med. 50, 1077–1088 10.1002/mrm.10609 14587019

[B7] Benjamini Y. Hochberg Y. (1995). Controlling the false discovery rate: a practical and powerful approach to multiple testing. J. R. Statist. Soc. B 57, 289–300

[B8] Bloodstein O. Ratner N. B. (2008). A Handbook on Stuttering. Clifton Park, NY: Thomson/Delmar Learning

[B9] Bohland J. W. Bullock D. Guenther F. H. (2010). Neural representations and mechanisms for the performance of simple speech sequences. J. Cogn. Neurosci. 22, 1504–1529 10.1162/jocn.2009.21306 19583476 PMC2937837

[B10] Cai S. Beal D. S. Ghosh S. S. Tiede M. K. Guenther F. H. Perkell J. S. (2012). Weak responses to auditory feedback perturbation during articulation in persons who stutter: evidence for abnormal auditory-motor transformation. PLoS ONE 7:e41830 10.1371/journal.pone.0041830 22911857 PMC3402433

[B11] Chang S. E. Erickson K. I. Ambrose N. G. Hasegawa-Johnson M. A. Ludlow C. L. (2008). Brain anatomy differences in childhood stuttering. Neuroimage 39, 1333–1344 10.1016/j.neuroimage.2007.09.067 18023366 PMC2731627

[B12] Chang S. E. Horwitz B. Ostuni J. Reynolds R. Ludlow C. L. (2011). Evidence of left inferior frontal-premotor structural and functional connectivity deficits in adults who stutter. Cereb. Cortex 21, 2507–2518 10.1093/cercor/bhr028 21471556 PMC3183422

[B13] Chang S. E. Zhu D. C. (2013). Neural network connectivity differences in children who stutter. Brain 136, 3709–3726 10.1093/brain/awt275 24131593 PMC3859219

[B14] Civier O. Tasko S. M. Guenther F. H. (2010). Overreliance on auditory feedback may lead to sound/syllable repetitions: simulations of stuttering and fluency-inducing conditions with a neural model of speech production. J. Fluency Disord. 35, 246–279 10.1016/j.jfludis.2010.05.002 20831971 PMC2939043

[B15] Connally E. L. Ward D. Howell P. Watkins K. E. (in press). Disrupted white matter in language and motor tracts in developmental stuttering. Brain Lang. Available online at: http://www.sciencedirect.com/science/article/pii/S0093934X13001119 10.1016/j.bandl.2013.05.013 23819900

[B16] Cykowski M. D. Fox P. T. Ingham R. J. Ingham J. C. Robin D. A. (2010). A study of the reproducibility and etiology of diffusion anisotropy differences in developmental stuttering: a potential role for impaired myelination. Neuroimage 52, 1495–1504 10.1016/j.neuroimage.2010.05.011 20471482 PMC4135434

[B17] Daliri A. Prokoenko R. A. Max L. (2013). Afferent and efferent aspects of mandibular sensorimotor control in adults who stutter. J. Speech Lang. Hear. Res. 56, 1774–1788 10.1044/1092-4388(2013/12-0134) 23816664 PMC3795963

[B18] De Nil L. F. Kroll R. M. Houle S. (2001). Functional neuroimaging of cerebellar activation during single word reading and verb generation in stuttering and nonstuttering adults. Neurosci. Lett. 302, 77–80 10.1016/S0304-3940(01)01671-8 11290391

[B19] Dyrby T. B. Sogaard L. V. Parker G. J. Alexander D. C. Lind N. M. Baare W. F. (2007). Validation of *in vitro* probabilistic tractography. Neuroimage 37, 1267–1277 10.1016/j.neuroimage.2007.06.022 17706434

[B20] Fischl B. (2012). FreeSurfer. Neuroimage 62, 774–781 10.1016/j.neuroimage.2012.01.021 22248573 PMC3685476

[B21] Giraud A. L. Neumann K. Bachoud-Levi A. C. von Gudenberg A. W. Euler H. A. Lanfermann H. (2008). Severity of dysfluency correlates with basal ganglia activity in persistent developmental stuttering. Brain Lang. 104, 190–199 10.1016/j.bandl.2007.04.005 17531310

[B22] Golfinopoulos E. Tourville J. A. Guenther F. H. (2010). The integration of large-scale neural network modeling and functional brain imaging in speech motor control. Neuroimage 52, 862–874 10.1016/j.neuroimage.2009.10.023 19837177 PMC2891349

[B23] Grabski K. Lamalle L. Vilain C. Schwartz J. L. Vallee N. Tropres I. (2012). Functional MRI assessment of orofacial articulators: neural correlates of lip, jaw, larynx, and tongue movements. Hum. Brain Mapp. 33, 2306–2321 10.1002/hbm.21363 21826760 PMC6870116

[B25] Guenther F. H. Ghosh S. S. Tourville J. A. (2006). Neural modeling and imaging of the cortical interactions underlying syllable production. Brain Lang. 96, 280–301 10.1016/j.bandl.2005.06.001 16040108 PMC1473986

[B26] Howell P. Au-Yeung J. (2002). The EXPLAN theory of fluency control applied to the diagnosis of stuttering, in Pathology and Therapy of Speech Disorders, ed Fava E. (Amsterdam: John Benjamins), 75–94

[B27] Jones D. K. (2010). Challenges and limitations of quantifying brain connectivity *in vivo* with diffusion MRI. Future Med. 2, 341–355

[B28] Kalinowski J. Armson J. Roland-Mieszkowski M. Stuart A. Gracco V. L. (1993). Effects of alterations in auditory feedback and speech rate on stuttering frequency. Lang. Speech 36, 1–16 8345771 10.1177/002383099303600101

[B29] Kell C. A. Neumann K. von Kriegstein K. Posenenske C. von Gudenberg A. W. Euler H. (2009). How the brain repairs stuttering. Brain 132, 2747–2760 10.1093/brain/awp185 19710179

[B30] Liu Z. Wang Y. Greig G. Gouttrard S. Tao R. Fletcher T. (2010). Quality control of diffusion weighted images, in Proceedings of the SPIE Medical Imaging (San Diego, CA). 10.1117/12.844748 PMC3864968 24353379

[B31] Loucks T. M. De Nil L. F. (2006). Oral kinesthetic deficit in adults who stutter: a target-accuracy study. J. Mot. Behav. 38, 238–246 10.3200/JMBR.38.3.238-247 16709563

[B33] Lu C. Peng D. Chen C. Ning N. Ding G. Li K. (2010). Altered effective connectivity and anomalous anatomy in the basal ganglia-thalamocortical circuit of stuttering speakers. Cortex 46, 49–67 10.1016/j.cortex.2009.02.017 19375076

[B34] Månsson H. (2000). Childhood stuttering: incidence and development. J. Fluency Disord. 25, 47–57 10.1016/S0094-730X(99)00023-6

[B35] Nieto-Castanon A. Ghosh S. S. Tourville J. A. Guenther F. H. (2003). Region of interest based analysis of functional imaging data. Neuroimage 19, 1303–1316 10.1016/S1053-8119(03)00188-5 12948689

[B36] Onozuka M. Fujita M. Watanabe K. Hirano Y. Niwa M. Nishiyama K. (2002). Mapping brain region activity during chewing: a functional magnetic resonance imaging study. J. Dent. Res. 81, 743–746 10.1177/154405910208101104 12407087

[B37] Onozuka M. Fujita M. Watanabe K. Hirano Y. Niwa M. Nishiyama K. (2003). Age-related changes in brain regional activity during chewing: a functional magnetic resonance imaging study. J. Dent. Res. 82, 657–660 10.1177/154405910308200817 12885854

[B38] Preibisch C. Raab P. Neumann K. Euler H. A. von Gudenberg A. W. Gall V. (2003). Event-related fMRI for the suppression of speech-associated artifacts in stuttering. Neuroimage 19, 1076–1084 10.1016/S1053-8119(03)00157-5 12880833

[B39] Reilly S. Onslow M. Packman A. Wake M. Bavin E. L. Prior M. (2009). Predicting stuttering onset by the age of 3 years: a prospective, community cohort study. Pediatrics 123, 270–277 10.1542/peds.2007-3219 19117892 PMC3879585

[B40] Riley G. D. (2008). SSI-4: Stuttering Severity Instrument. (Austin, TX: PRO-ED).

[B41] Rubinov M. Sporns O. (2010). Complex network measures of brain connectivity: uses and interpretations. Neuroimage 52, 1059–1069 10.1016/j.neuroimage.2009.10.003 19819337

[B42] Scholz J. Klein M. C. Behrens T. E. Johansen-Berg H. (2009). Training induces changes in white-matter architecture. Nat. Neurosci. 12, 1370–1371 10.1038/nn.2412 19820707 PMC2770457

[B43] Smith S. M. Jenkinson M. Johansen-Berg H. Rueckert D. Nichols T. E. Mackay C. E. (2006). Tract-based spatial statistics: voxelwise analysis of multi-subject diffusion data. Neuroimage 31, 1487–1505 10.1016/j.neuroimage.2006.02.024 16624579

[B44] Smith S. M. Nichols T. E. (2009). Threshold-free cluster enhancement: addressing problems of smoothing, threshold dependence and localisation in cluster inference. Neuroimage 44, 83–98 10.1016/j.neuroimage.2008.03.061 18501637

[B45] Sommer M. Koch M. A. Paulus W. Weiller C. Buchel C. (2002). Disconnection of speech-relevant brain areas in persistent developmental stuttering. Lancet 360, 380–383 10.1016/S0140-6736(02)09610-1 12241779

[B46] Takahashi T. Miyamoto T. Terao A. Yokoyama A. (2007). Cerebral activation related to the control of mastication during changes in food hardness. Neuroscience 145, 791–794 10.1016/j.neuroscience.2006.12.044 17320301

[B47] Takai O. Brown S. Liotti M. (2010). Representation of the speech effectors in the human motor cortex: somatotopy or overlap? Brain Lang. 113, 39–44 10.1016/j.bandl.2010.01.008 20171727

[B55] Tourville J. A. Reilly K. J. Guenther F. H. (2008). Neural mechanisms underlying auditory feedback control of speech. Neuroimage 39, 1429–1443 10.1016/j.neuroimage.2007.09.054 18035557 PMC3658624

[B48] Toyomura A. Fujii T. Kuriki S. (2011). Effect of external auditory pacing on the neural activity of stuttering speakers. Neuroimage 57, 1507–1516 10.1016/j.neuroimage.2011.05.039 21624474

[B49] Watkins K. E. Smith S. M. Davis S. Howell P. (2008). Structural and functional abnormalities of the motor system in developmental stuttering. Brain 131, 50–59 10.1093/brain/awm241 17928317 PMC2492392

[B50] Yamada M. Momoshima S. Masutani Y. Fujiyoshi K. Abe O. Nakamura M. (2008). Diffusion-tensor neuronal fiber tractography and manganese-enhanced mr imaging of primate visual pathway in the common marmoset: preliminary results. Radiology 249, 855–864 10.1148/radiol.2493072141 19011185

[B52] Zalesky A. Fornito A. Bullmore E. T. (2010). Network-based statistic: identifying differences in brain networks. Neuroimage 53, 1197–1207 10.1016/j.neuroimage.2010.06.041 20600983

[B53] Zalesky A. Fornito A. Seal M. L. Cocchi L. Westin C. F. Bullmore E. T. (2011). Disrupted axonal fiber connectivity in schizophrenia. Biol. Psychiatry 69, 80–89 10.1016/j.biopsych.2010.08.022 21035793 PMC4881385

[B54] Zatorre R. J. Fields R. D. Johansen-Berg H. (2012). Plasticity in gray and white: neuroimaging changes in brain structure during learning. Nat. Neurosci. 15, 528–536 10.1038/nn.3045 22426254 PMC3660656

