# Supplementary material for: Diffusion imaging of cerebral white matter in persons who stutter: evidence for network-level anomalies
Source: Front Hum Neurosci. 2014 Feb 11;8:54. doi: 10.3389/fnhum.2014.00054 (PMC3920071; doi:10.3389/fnhum.2014.00054)
Supplement: Supplementary file 1 [file DataSheet1.PDF]

Supplemental Materials

SpeechLabel parcellation: The cortical labeling process

Labeling was initially applied automatically to each cortical surface using an atlas that was constructed using standard FreeSurfer tools (Fischl et al., 2012; Tourville and Guenther, 2012). The SpeechLabel atlas was trained on a set of cortical surfaces from 17 neurologically normal participants (separate from the current study) that were manually edited to conform to the SpeechLabel protocol by an expert in MRI-based neuroanatomical labeling with 16 years experience labeling cortical structures (J.A.T.) Following the initial parcellation of cortical surfaces from each participant in the present study using the SpeechLabel atlas, each labeled surface was reviewed and manually edited by another experienced labeler and inspected by J.A.T. to ensure accuracy and consistency across hemispheres and participants.

SpeechLabel parcellation: The speech network

The determination of the speech-network subset was based on an fMRI study in which the task was oral reading of visually presented sentences (Golfinopoulos, 2012). Fourteen healthy participants participated in that study. The BOLD activations during the oral reading were contrasted with those under a silent baseline condition. For second (group) level analysis, first-level contrasts in the left hemisphere were mapped onto the FreeSurfer's *fsaverage* template, which was itself parcellated by SpeechLabel. The second-level analysis used cluster-level correction for multiple comparisons based on a vertex p-value threshold of 0.001 and a cluster-size p-value threshold of 0.025 (Monte Carlo simulation, 10000 iterations). A SpeechLabel region was included in the speech network if and only if it had any overlap with the significant

clusters. The same set of 28 ROIs was used to analyze the white-matter properties in both hemispheres. Table S1 below gives a detailed description of the 28 speech ROIs, including their anatomical definitions and approximate Brodmann areas.

This speech network is largely consistent with current theories on the neural bases of speech production. All the cortical regions that play roles in the DIVA model of speech motor control (Guenther et al., 2006, Golfinopoulos et al., 2010) and the GODIVA model of unit sequencing in speech production (Bohland et al. 2010) are included in this 28-ROI subset. Of the ROIs in the subset that have not been assigned explicit roles in these models, anterior cingulate gyrus (aCGg) is known to be involved in phonation (e.g., Jürgens and von Cramon, 1982); parts of the insula show activation under speech production tasks (e.g., Ackermann and Riecker, 2010; Bohland et al., 2006; Ghosh et al., 2008); parts of the anterior superior temporal gyrus (aSTg) and planum polare (PP) has also been observed to show activation during certain speech production tasks (e.g., Ghosh et al., 2008; Tourville et al., 2008; Golfinopoulos et al., 2011).

Table S1. List of speech-network regions of interest (ROIs) in the SpeechLabel parcellation.

**Abbreviated name of ROI**	**Full name of ROI**	**Approximate Brodmann area**	**Anatomical definition**
aCGg	Anterior cingulate gyrus	24	portion of the cingulate gyrus that lies anterior to the genu of the corpus callosum
aCO	Central operculum, anterior division	4 & 43	Portion of the dorsal bank of the Sylvian fissure that is bounded anteriorly by the rostro-lateral limit of the precentral sulcus and posteriorly by the rostro-lateral limit of the central sulcus
aFO	Frontal operculum, anterior division	45	portion of the dorsal bank of the Sylvian fissure that is bounded anteriorly by the anterior limit of the insular cortex and posteriorly by the anterior ascending ramus of the Sylvian fissure
aINS	Anterior insula	--	portion of the insular cortex that lies dorso-rostral to central sulcus of the insula
aSMg	Supramarginal gyrus, anterior division	40	Portion of parietal cortex that lies lateral to the intraparietal sulcus; it is bounded anteriorly by the postcentral sulcus and posteriorly by the caudo-posterior limit of the posterior ramus of the Sylvian

			fissure.
aSTg	Superior temporal gyrus, anterior division	22	Lateral external portion of the superior temporal gyrus bounded anteriorly by the anterior limit of the superior temporal sulcus and posteriorly by the anterior limit of Heschl's sulcus; it is bounded dorsally by the lateral margin of the ventral bank of the Sylvian fissure and ventrally by the lateral margin of the superior temporal sulcus
dIFo	Inferior frontal gyrus pars opercularis, dorsal division	44	portion of the inferior frontal gyrus that extends from the dorsal-ventral midpoint of the gyrus dorsally to the lateral margin of the ventral bank of the inferior frontal sulcus; it is bounded anteriorly by the anterior ascending ramus of the Sylvian fissure and posteriorly by the precentral sulcus
dMC	Primary motor cortex, dorsal division	4	Caudal half of the precentral gyrus that lies dorso-medial to the motor hand knob
dSC	Primary somatosensory cortex, dorsal division	1, 2 & 3	Portion of the postcentral gyrus that lies dorso-medial to the junction of the postcentral sulcus and the intraparietal sulcus
Hg	Heschl's gyrus	41	Transverse gyrus on the ventral bank of the Sylvian fissure (supratemporal plane) that lies between the first transverse temporal sulcus and Heschl's sulcus
midMC	Primary motor cortex, middle division	4	Caudal half of the precentral gyrus that lies dorso-medial to the junction of the precentral sulcus and the inferior frontal sulcus and ventro-lateral to the anatomical marker for the hand motor representation, commonly referred to as the "motor hand knob" (e.g., Yousry et al., 1997)
midPMC	Lateral premotor cortex, middle division	6	Rostral half of the precentral gyrus that lies dorso-medial to the junction of the precentral sulcus and the inferior frontal sulcus and ventro-lateral to the motor hand knob
pCO	Posterior central operculum	1, 2, 3 & 43	Portion of the dorsal bank of the Sylvian fissure that is bounded anteriorly by the rostro-lateral limit of the central sulcus and posteriorly by the rostro-lateral limit of the postcentral sulcus
pdPMC	Lateral premotor cortex, posterior dorsal division	6	Rostral half of the precentral gyrus that lies dorso-medial to the motor hand knob
pdSTs	Superior temporal sulcus, posterior dorsal division	22	Dorsal bank of the superior temporal sulcus, see pSTg for anterior and posterior bounds
pFO	Posterior frontal operculum	44	portion of the dorsal bank of the Sylvian fissure that is bounded anteriorly by the anterior ascending ramus of the Sylvian fissure posteriorly by the rostro-lateral limit of the precentral sulcus
pIFs	Posterior inferior frontal sulcus	44 & 46	portion of the inferior frontal sulcus that is bounded anteriorly by the rostro-medial limit of the anterior ascending ramus of the Sylvian fissure and posteriorly by the precentral sulcus
pINS	Posterior insula	--	portion of the insular cortex that lies ventro-causal to central sulcus of the insula
PO	Parietal operculum	40	Portion of the dorsal bank of the Sylvian fissure that is bounded anteriorly by the rostro-lateral limit of the

			postcentral sulcus and posteriorly by the posterior limit of the posterior ascending ramus of the Sylvian fissure
PP	Planum polare	38	Portion of the ventral bank of the Sylvian fissure bounded anteriorly by the fronto-temporal junction and posteriorly by the first transverse temporal sulcus
preSMA	Pre-supplementary motor area	6	portion of medial prefrontal cortex extending from the dorsal hemispheric margin ventrally to cingulate sulcus; it is bounded anteriorly by the genu of the corpus callosum and posteriorly by the decussation of the anterior commissure
pSTg	Superior temporal gyrus, posterior division	42 & 22	Lateral external portion of the superior temporal gyrus bounded by the anterior limit of Heschl's sulcus anteriorly and the posterior ascending ramus of the superior temporal sulcus or the primary intermediate sulcus posteriorly; it is bounded dorsally by the lateral margin of the ventral bank of the Sylvian fissure and ventrally by the lateral margin of the superior temporal sulcus
PT	Planum temporale	42 & 22	Portion of ventral bank of the Sylvian fissure bounded by the Heschl's sulcus anteriorly and the posterior limit of the posterior ascending ramus of the Sylvian fissure
SMA	Supplementary motor area (proper)	6	portion of medial prefrontal cortex extending from the dorsal hemispheric margin ventrally to cingulate sulcus; it is bounded anteriorly by the decussation of the anterior commissure and posteriorly by the caudo-medial limit of the precentral sulcus
vIFo	Inferior frontal gyrus pars opercularis, ventral division	44	portion of the inferior frontal gyrus that extends from the dorsal-ventral midpoint of the gyrus ventrally to the lateral margin of the dorsal bank of the Sylvian fissure; it is bounded anteriorly by the anterior ascending ramus of the Sylvian fissure and posteriorly by the precentral sulcus
vMC	Primary motor cortex, ventral division	4	Caudal half of the precentral gyrus that lies ventro-lateral to the junction of the precentral sulcus and the inferior frontal sulcus
vPMC	Lateral premotor cortex, ventral division	6	Rostral half of the precentral gyrus that lies ventro-lateral to the junction of the precentral sulcus and the inferior frontal sulcus
vSC	Primary somatosensory cortex, ventral division	1, 2 & 3	Portion of the postcentral gyrus that lies ventro-lateral to the junction of the postcentral sulcus and the intraparietal sulcus

Table S2. Full list of abbreviations.

Abbreviation	Full name	Abbreviation	Full name
**aCGg**	anterior cingulate gyrus	**aCO**	central operculum, anterior division
**adPMC**	premotor cortex, anterior dorsal division	**adSTs**	superior temporal sulcus, anterior dorsal division
**AF**	arcuate fasciculus	**aFO**	frontal operculum, anterior division
**Ag**	angular gyrus	**aIFs**	inferior frontal sulcus, anterior division
**aINS**	anterior insula	**aMFg**	middle frontal gyrus, anterior division
**aMTg**	middle temporal gyrus, anterior division	**aPH**	parahippocampal gyrus, anterior division
**aSMg**	supramarginal gyrus, anterior division	**aSTg**	superior temporal gyrus, anterior division
**avSTs**	superior temporal gyrus, anterior ventral division	**AWS**	adults who stutter
**BA**	Brodmann area	**CST**	corticospinal tract
**CWS**	children who stutter	**dIFo**	inferior frontal gyrus pars opercularis, dorsal division
**dSC**	somatosensory cortex, dorsal division	**dMC**	motor cortex, dorsal division
**DWI**	diffusion-weighted imaging	**FA**	fractional anisotropy
**FDT**	FMRIB's diffusion toolbox	**FMC**	frontal medial cortex
**FOC**	frontal orbital cortex	**FP**	frontal pole
**FWE**	family-wise error	**GM**	gray matter
**Hg**	Heschl's gyrus	**IFG**	inferior frontal gyrus;
**IFo**	inferior frontal gyrus, pars opercularis	**ITOg**	inferior temporal occipital gyrus
**Lg**	Lingual gyrus	**mdPMC**	premotor cortex, middle dorsal division
**midCGg**	cingulate gyrus, middle division	**midMC**	motor cortex, middle division
**midPMC**	premotor cortex, middle division	**MNI**	Montreal Neurological Institute
**MTOg**	middle temporal occipital gyrus	**NBS**	network-based statistic
**OC**	occipital cortex	**pCGg**	cingulate gyrus, posterior division
**PCN**	Precuneus	**pdPMC**	premotor cortex, posterior dorsal division
**pdSTs**	superior temporal sulcus, posterior dorsal division	**PFS**	person(s) with fluent speech
**pIFs**	posterior inferior frontal gyrus	**pINS**	posterior insula
**pITg**	inferior temporal gyrus, posterior division	**pMFg**	posterior middle frontal gyrus
**pMTg**	middle temporal gyrus, posterior division	**pFO**	frontal operculum, posterior division
**pPH**	parahippocampal gyrus, posterior division	**preSMA**	pre-supplementary motor area
**pvSTs**	superior temporal sulcus, posterior dorsal division	**PWS**	person(s) who stutter
**PO**	parietal operculum	**PP**	planum temporale
**pSMg**	supramarginal gyrus, posterior division	**pSTg**	superior temporal gyrus, posterior division
**PT**	planum temporale	**ROI**	region of interest
**SFG**	superior frontal gyrus	**SLF**	superior longitudinal fasciculus
**SLP**	speech language pathologist	**SMA**	supplementary motor area
**SMg**	Supramarginal gyrus	**SPL**	superior parietal lobule
**SSI**	stuttering severity instrument	**TE**	echo time
**TFCE**	threshold-free cluster enhancement	**TP**	temporal pole

**TR**	repetition time	**TBSS**	tract-based spatial statistic
**vIFo**	inferior frontal gyrus pars opercularis, ventral division	**vMC**	motor cortex, ventral division
**vPMC**	premotor cortex, ventral division	**vSC**	somatosensory cortex, ventral division
**WM**	white matter		

Analysis of regional average diffusion tensor measures

Tables S3 – S6 below summarize significant findings ($p < 0.05$, uncorrected) from analyses on the ROI averages of four basic tensor measures of WM diffusivity, including fractional anisotropy (FA, detailed in main text Sect. 3.3), mean diffusivity (MD), axial diffusivity (AD) and radial diffusivity (RD).

Table S3: Analysis of regional average fractional anisotropy (FA)

**Between-group differences**			
ROI name	*t-value*	*p-value*	*Direction of difference*
L dIFo	-2.444	0.0197	PWS < PFS
L pdSTs	-2.058	0.0470	PWS < PFS
L midMC	-2.049	0.0480	PWS < PFS
R pdSTs	-2.640	0.0123	PWS < PFS
L pMTg	2.510	0.0168	PWS > PFS

**Correlation with severity (SSI-4) score in PWS**		
ROI Name	*Correlation coefficient (R)*	*p-value*
L dMC	-0.530	0.0161
L vMC	-0.514	0.0203
L midMC	-0.542	0.0135
R dMC	-0.465	0.0390
L pCO	0.596	0.0055
L PO	0.499	0.0251
L MTOg	0.478	0.0332

Table S4: Analysis of regional average mean diffusivity (MD)

**Between-group differences**			
ROI name	*t-value*	*p-value*	*Direction of difference*
(None)			

**Correlation with severity (SSI-4) score in PWS**		
ROI Name	*Correlation coefficient (R)*	*p-value*
L pSTg	-0.566	0.0093
L adSTs	-0.527	0.0169
L pMTg	-0.552	0.0116

L aMFg	-0.568	0.0090

Table S5: Analysis of regional average axial diffusivity (AD)

**Between-group differences**			
ROI name	*t-value*	*p-value*	*Direction of difference*
L pdSTs	-2.187	0.0355	PWS < PFS
L ITOg	-2.132	0.0401	PWS < PFS

**Correlation with severity (SSI-4) score in PWS**		
ROI Name	*Correlation coefficient (R)*	*p-value*
L pSTg	-0.530	0.0162
L pMTg	-0.476	0.0339
L aMFg	-0.611	0.0042
L midMC	-0.507	0.0224
R dMC	-0.496	0.0260
R FOC	-0.548	0.0124

Table S6: Analysis of regional average radial diffusivity (RD)

**Between-group differences**			
ROI name	*t-value*	*p-value*	*Direction of difference*
(None)			

**Correlation with severity (SSI-4) score in PWS**		
ROI Name	*Correlation coefficient (R)*	*p-value*
L pSTg	-0.467	0.0381
L pMTg	-0.529	0.0166
L MTOg	-0.515	0.0202

References

Ackermann H, Riecker A. 2010. The contribution(s) of the insula to speech production: a review of the clinical and functional imaging literature. Brain Struct. Funct. 214: 419-433.

Bohland JW, Guenther FH. 2006. An fMRI investigation of syllable sequence production. Neuroimage. 32: 821-841.

Fischl B. 2012. FreeSurfer. Neuroimage. 62: 774-781.

Ghosh SS, Tourville JA, Guenther FH. 2008. A neuroimaging study of premotor lateralization and cerebellar involvement in the production of phonemes and syllables. J Speech Lang Hear Res. 51: 1183-1202.

Golfinopoulos E. 2012. From Prosodic Structure to Acoustic Saliency: An FMRI Investigation of Speech Rate, Clarity, and Emphasis. Unpublished doctoral dissertation. Department of Cognitive and Neural Systems, Boston University, Boston, MA, USA.

Golfinopoulos E, Tourville JA, Bohland JW, Ghosh SS, Nieto-Castanon A, Guenther FH. 2011. fMRI investigation of unexpected somatosensory feedback perturbation during speech. Neuroimage. 55: 1324-1338.

Jürgens U, von Cramon D. 1982. On the role of the anterior cingulate cortex in phonation: a case report. Brain Lang, 15: 234-248.

Tourville JA, Guenther FH. 2012. Automatic cortical labeling system for neuroimaging studies of normal and disordered speech. 42nd Annual Meeting for the Society for Neuroscience. New Orleans, LA.

Tourville JA, Reilly KJ, Guenther FH. 2008. Neural mechanisms underlying auditory feedback control of speech. Neuroimage, 39: 1429-1443.
